# Supplementary material for: Genetic variant effects on gene expression in human pancreatic islets and their implications for T2D
Source: Nat Commun. 2020 Sep 30;11:4912. doi: 10.1038/s41467-020-18581-8 (PMC7528108; doi:10.1038/s41467-020-18581-8)
Supplement: Supplementary file 1 — Supplementary Information [file 41467_2020_18581_MOESM1_ESM.pdf]

Supplementary Information

Genetic variant effects on gene expression in human pancreatic islets and their implications for T2D. Viñuela, Varshney, van de Bunt, Prasad, et al.

Index:

Supplementary Tables ……….2

Supplementary Discussion ……………………………………………………………………………………………………. 11

Supplementary Figures ……. 13

Supplementary References …………………………………………………………………………………………………….41

Supplementary Tables

Supplementary Table 1 | Islet eSNP overlap and enrichment in islet chromatin states. Input included: 1% FDR eQTL SNPs, filtered for MAF >= 0.2, LD pruned to retain SNPs with LD r2 <0.8. Enrichment was run using GREGOR tool which reports one-sided P values for enrichment.

Cell	Chromatin State	Overlap	Expected Overlap	Pval	Fold Enrichment
**Islets**	1 Active TSS	646	168.4285695	5.5163E-206	3.835453819
**Islets**	10 Active enhancer 2	646	373.7879896	6.36051E-45	1.728252426
**Islets**	11 Weak enhancer	107	55.45538991	1.32109E-10	1.929478815
**Islets**	14 Bivalent poised TSS	8	8.943588041	0.670992632	0.894495583
**Islets**	16 Repressed polycomb	42	64.06888459	0.998787342	0.655544423
**Islets**	17 Weak repressed polycomb	567	980.5626747	1	0.578239428
**Islets**	18 Quiescent low signal	814	1378.784788	1	0.59037495
**Islets**	2 Weak TSS	220	91.00643384	3.5609E-34	2.417411503
**Islets**	3 Flanking TSS	31	9.69543573	2.62495E-08	3.197380795
**Islets**	5 Strong transcription	533	226.6297053	3.4569E-82	2.351854093
**Islets**	6 Weak transcription	2212	1508.922102	7.2111E-124	1.465947114
**Islets**	8 Genic enhancer	19	8.659541481	0.001354008	2.194111552
**Islets**	9 Active enhancer 1	29	14.55833157	0.000483652	1.991986503
**Islets**	stretch enhancer	279	178.2337579	2.76187E-13	1.565360026

Supplementary Table 2 | T2D GWAS overlap and enrichment in Islet chromatin states. Input included: T2D GWAS lead SNPs LD pruned to retain SNPs with LD r2 <0.2. Enrichment was run using GREGOR tool which reports one-sided P values for enrichment.

Trait	Tissue	Chromatin State	Overlap	Expected Overlap	Pval	Fold Enrichment
**T2D**	Islets	1 Active TSS	83	36.75071553	5.98278E-15	2.258459429
**T2D**	Islets	10 Active enhancer 2	148	68.08416513	4.19572E-25	2.173780052
**T2D**	Islets	11 Weak enhancer	33	15.02336156	7.49315E-06	2.196578967
**T2D**	Islets	14 Bivalent poised TSS	4	2.897756572	0.329040071	1.3803782
**T2D**	Islets	16 Repressed polycomb	14	14.48682595	0.590580855	0.966395265
**T2D**	Islets	17 Weak repressed polycomb	97	130.7152207	0.999946431	0.742071195
**T2D**	Islets	18 Quiescent low signal	142	176.804994	0.999953803	0.803144735
**T2D**	Islets	2 Weak TSS	41	21.91059464	3.22446E-05	1.871240862
**T2D**	Islets	3 Flanking TSS	4	2.616307877	0.265935301	1.528872055
**T2D**	Islets	5 Strong transcription	54	36.73682051	0.001115897	1.469914904
**T2D**	Islets	6 Weak transcription	231	164.6976275	9.78215E-14	1.402570295
**T2D**	Islets	8 Genic enhancer	2	1.664686213	0.500115358	1.201427623
**T2D**	Islets	9 Active enhancer 1	10	4.005432849	0.006917117	2.496609075
**T2D**	Islets	stretch enhancer	84	31.15435816	1.52255E-18	2.696251984

Supplementary Table 3 | Islet eSNP overlap and enrichment in ATAC-seq peaks within Islet chromatin states. Input included: 1% FDR eQTL SNPs, filtered for MAF >= 0.2, LD pruned to retain SNPs with LD r2 <0.8. Enrichment was run using GREGOR tool which reports one-sided P values for enrichment. GREGOR LD R2 threshold = 0.99.

Tissue	Chromatin State	Overlap	Expected Overlap	Pval
**Islets**	1_Active_TSS	529	126.7956399	2.60E-181
**Islets**	10_Active_enhancer_2	243	127.6952763	3.96E-22
**Islets**	11_Weak_enhancer	8	6.679353733	0.35314905
**Islets**	14_Bivalent_poised_TSS	3	3.166393089	0.61434421
**Islets**	16_Repressed_polycomb	2	2.211965007	0.65024766
**Islets**	17_Weak_repressed_polycomb	23	22.49119298	0.48560423
**Islets**	18_Quiescent_low_signal	47	45.79505585	0.44852934
**Islets**	2_Weak_TSS	78	27.00452726	1.79E-16
**Islets**	3_Flanking_TSS	15	4.329971958	4.04E-05
**Islets**	5_Strong_transcription	3	1.763515067	0.25810763
**Islets**	6_Weak_transcription	99	73.82695003	0.0021955
**Islets**	8_Genic_enhancer	1	0.374846627	0.31699116
**Islets**	9_Active_enhancer_1	7	6.054394149	0.40246364
**Islets**	all atac-seq_peaks	905	390.0891585	4.59E-142
**Islets**	stretchEnhancer	93	56.19877741	2.85E-06
**Islets**	typicalEnhancer	35	17.33145902	9.25E-05

Supplementary Table 4 | Islet eSNP enrichment in TF footprint-motifs and motif directionality. Motif by name with their enrichment and directionality. eQTL fold enrichment: Islet eQTL Fold enrichment in footprint motifs. eQTL enrichment Pvalue: Pvalue for enrichment of eQTL in footprint motifs. Total eQTL: Total eQTL overlaps with footprint motifs at positions with motif information content >= 0.7. Also, either REF or ALT allele should be the most preferred based in the motif. Motif directionality: Fraction of eQTL where most preferred base is associated with increased target gene expression. HepG2 activity: MPRA Activity score in HepG2. K562 activity: MPRA Activity score in K562. Included the binomial test Pvalue and the binomial test significance.

Motif	eQTL Fold enrichment	eQTL enrichment Pvalue	Total eQTL	Motif directionality	HepG2 activity	K562 activity	Binomial test P	Binomial test significance
**AP1_known3**	3.554	0.000	5.000	0.600	0.560	0.675	1.000	Not significant
**AP1_known6**	3.206	0.000	11.000	0.909	0.614	0.676	0.012	Significant FDR<10%
**ARNT_1**	3.773	0.000	14.000	0.500	0.350	0.420	1.000	Not significant
**ATF3_disc2**	3.784	0.000	78.000	0.538	1.046	1.143	0.572	Not significant
**ATF3_known4**	2.869	0.000	12.000	0.500	0.697	0.601	1.000	Not significant
**BCL_disc1**	4.232	0.000	15.000	0.867	1.042	1.106	0.007	Significant FDR<10%
**BHLHE40_known2**	3.770	0.000	14.000	0.357	0.427	0.384	0.424	Not significant
**CREB3L1_4**	3.389	0.000	16.000	0.688	0.600	0.370	0.210	Not significant
**CTCFL_disc1**	2.990	0.000	54.000	0.500	0.235	0.339	1.000	Not significant
**CTCF_disc1**	2.622	0.000	20.000	0.400	0.088	0.182	0.503	Not significant
**CTCF_disc2**	3.004	0.000	38.000	0.395	0.239	0.323	0.256	Not significant

**CTCF_known2**	2.811	0.000	28.000	0.464	0.158	0.282	0.851	Not significant
**E2F_disc2**	3.376	0.000	28.000	0.643	1.331	1.429	0.185	Not significant
**E2F_known18**	4.762	0.000	30.000	0.333	0.289	0.924	0.099	Not significant
**EGR1_disc2**	3.502	0.000	19.000	0.737	0.587	0.618	0.064	Not significant
**EGR1_disc3**	3.824	0.000	45.000	0.644	1.261	1.397	0.072	Not significant
**EGR1_disc5**	4.045	0.000	20.000	0.700	0.290	0.369	0.115	Not significant
**EGR1_disc7**	3.695	0.000	43.000	0.465	0.828	0.859	0.542	Not significant
**EGR1_known1**	3.337	0.000	44.000	0.568	0.147	0.331	0.451	Not significant
**EGR1_known5**	3.202	0.000	51.000	0.627	0.266	0.500	0.092	Not significant
**EGR1_known8**	3.565	0.000	66.000	0.500	0.241	0.410	1.000	Not significant
**EGR1_known9**	3.624	0.000	50.000	0.560	0.289	0.397	0.480	Not significant
**EGR3_3**	3.826	0.000	58.000	0.500	0.242	0.399	1.000	Not significant
**EHF_1**	3.194	0.000	10.000	0.900	1.084	1.202	0.021	Nominally significant (P<0.05)
**ELF1_disc1**	4.135	0.000	12.000	0.917	1.476	1.572	0.006	Significant FDR<10%
**ELF2_1**	2.709	0.000	28.000	0.571	0.166	0.157	0.572	Not significant
**ELF3_3**	3.614	0.000	13.000	0.769	0.728	0.751	0.092	Not significant
**ELF4_1**	3.603	0.000	11.000	0.909	1.000	1.095	0.012	Significant FDR<10%
**ELF5_3**	4.030	0.000	7.000	0.714	0.756	0.805	0.453	Not significant
**ELF5_4**	3.585	0.000	5.000	0.600	0.750	0.810	1.000	Not significant
**ELK3_1**	4.619	0.000	12.000	1.000	1.675	1.676	0.000	Significant FDR<10%
**ELK3_2**	4.362	0.000	12.000	0.917	1.675	1.676	0.006	Significant FDR<10%
**ELK4_1**	7.220	0.000	15.000	0.733	1.655	1.573	0.118	Not significant
**ELK4_2**	5.984	0.000	13.000	0.846	1.678	1.663	0.022	Nominally significant (P<0.05)
**ERG_1**	5.537	0.000	11.000	0.818	1.592	1.620	0.065	Not significant
**ERG_3**	5.435	0.000	14.000	0.786	1.597	1.582	0.057	Not significant
**ERG_4**	4.541	0.000	11.000	0.545	0.458	0.370	1.000	Not significant
**ETS_1**	2.965	0.000	20.000	0.750	0.267	0.280	0.041	Nominally significant (P<0.05)
**ETS_2**	3.487	0.000	13.000	0.692	0.132	0.215	0.267	Not significant
**ETS_known1**	2.885	0.000	16.000	0.688	0.321	0.299	0.210	Not significant
**ETS_known10**	4.241	0.000	12.000	0.917	1.401	1.259	0.006	Significant FDR<10%
**ETS_known11**	4.858	0.000	12.000	1.000	1.745	1.732	0.000	Significant FDR<10%
**ETS_known13**	4.103	0.000	22.000	0.545	0.801	0.992	0.832	Not significant
**ETS_known14**	5.656	0.000	15.000	0.867	1.674	1.693	0.007	Significant FDR<10%
**ETS_known15**	3.407	0.000	19.000	0.474	0.387	0.487	1.000	Not significant
**ETS_known16**	6.027	0.000	12.000	0.833	1.641	1.660	0.039	Nominally significant (P<0.05)
**ETS_known18**	3.836	0.000	11.000	0.909	1.609	1.670	0.012	Significant FDR<10%
**ETS_known2**	4.342	0.000	17.000	0.765	1.195	1.164	0.049	Nominally significant (P<0.05)
**ETS_known3**	4.429	0.000	13.000	0.769	0.692	0.633	0.092	Not significant
**ETS_known6**	3.467	0.000	39.000	0.641	0.749	0.841	0.108	Not significant
**ETS_known7**	4.047	0.000	15.000	0.933	0.852	0.963	0.007	Significant FDR<10%
**ETS_known9**	4.358	0.000	21.000	0.857	1.763	1.989	0.001	Significant FDR<10%
**ETV1_1**	5.359	0.000	12.000	0.917	1.626	1.619	0.006	Significant FDR<10%
**ETV4_2**	5.688	0.000	13.000	0.923	1.578	1.621	0.003	Significant FDR<10%
**ETV5_1**	5.980	0.000	15.000	0.867	1.548	1.560	0.007	Significant FDR<10%
**ETV6_1**	3.670	0.000	29.000	0.759	0.782	0.972	0.008	Significant FDR<10%
**ETV6_2**	4.714	0.000	14.000	0.857	1.031	1.293	0.013	Significant FDR<10%

**FEV_2**	4.980	0.000	10.000	1.000	1.572	1.597	0.002	Significant FDR<10%
**FLI1_1**	6.135	0.000	13.000	0.846	1.576	1.618	0.022	Nominally significant (P<0.05)
**FLI1_3**	5.301	0.000	11.000	0.818	1.678	1.663	0.065	Not significant
**FLI1_4**	4.904	0.000	12.000	0.583	0.533	0.512	0.774	Not significant
**GATA_disc3**	2.538	0.000	14.000	0.286	0.151	0.143	0.180	Not significant
**GCM_1**	3.493	0.000	10.000	0.400	0.304	0.282	0.754	Not significant
**HDAC2_disc3**	2.692	0.000	41.000	0.463	-0.627	-0.334	0.755	Not significant
**HDAC2_disc4**	3.678	0.000	61.000	0.508	0.836	1.014	1.000	Not significant
**HINFP_2**	3.428	0.000	36.000	0.611	0.479	0.480	0.243	Not significant
**HINFP_3**	4.151	0.000	93.000	0.527	0.370	0.345	0.679	Not significant
**KLF12_2**	4.210	0.000	66.000	0.591	0.193	0.515	0.175	Not significant
**KLF14_1**	3.595	0.000	80.000	0.550	0.293	0.596	0.434	Not significant
**MXI1_known1**	3.639	0.000	11.000	0.545	0.233	0.194	1.000	Not significant
**MYB_1**	3.939	0.000	15.000	0.667	0.251	0.343	0.302	Not significant
**MYC_known11**	3.118	0.000	11.000	0.727	0.307	0.264	0.227	Not significant
**MYC_known19**	3.051	0.000	22.000	0.455	0.133	0.186	0.523	Not significant
**MYC_known6**	2.921	0.000	10.000	0.600	0.467	0.556	0.754	Not significant
**MYOD1_3**	2.923	0.000	27.000	0.370	-0.118	-0.099	0.248	Not significant
**NR2C2_disc3**	3.566	0.000	33.000	0.394	0.167	0.093	0.296	Not significant
**NRF1_disc1**	3.403	0.000	88.000	0.602	1.348	1.442	0.069	Not significant
**NRF1_known1**	3.356	0.000	35.000	0.514	1.336	1.432	1.000	Not significant
**NRF1_known2**	3.384	0.000	29.000	0.552	1.393	1.494	0.711	Not significant
**PAX2_4**	3.642	0.000	18.000	0.667	0.549	0.731	0.238	Not significant
**PAX5_disc4**	2.903	0.000	22.000	0.591	0.245	0.304	0.523	Not significant
**PAX5_disc5**	3.326	0.000	90.000	0.467	0.468	0.672	0.598	Not significant
**PAX5_known5**	3.593	0.000	18.000	0.667	0.598	0.731	0.238	Not significant
**PBX3_disc1**	5.311	0.000	8.000	0.625	0.467	0.619	0.727	Not significant
**PBX3_disc3**	3.153	0.000	39.000	0.487	0.271	0.265	1.000	Not significant
**RAD21_disc1**	2.990	0.000	49.000	0.429	0.121	0.205	0.392	Not significant
**REST_disc1**	2.944	0.000	35.000	0.457	-0.917	-0.491	0.500	Not significant
**REST_disc3**	3.060	0.000	31.000	0.355	-0.457	-0.278	0.150	Not significant
**REST_known3**	2.913	0.000	73.000	0.507	-0.521	-0.207	1.000	Not significant
**REST_known4**	2.708	0.000	51.000	0.569	-0.752	-0.450	0.401	Not significant
**SETDB1_disc1**	3.570	0.000	32.000	0.656	0.529	0.488	0.110	Not significant
**SIN3A_disc1**	3.251	0.000	24.000	0.667	-0.877	-0.455	0.152	Not significant
**SP2_disc2**	3.397	0.000	20.000	0.400	0.438	0.480	0.503	Not significant
**SP2_disc3**	3.000	0.000	37.000	0.486	0.357	0.529	1.000	Not significant
**SPDEF_5**	4.594	0.000	12.000	0.750	1.023	0.996	0.146	Not significant
**SREBP_disc1**	3.625	0.000	25.000	0.640	-0.216	0.001	0.230	Not significant
**STAT_known2**	3.165	0.000	12.000	0.583	0.451	0.556	0.774	Not significant
**TATA_disc8**	3.277	0.000	51.000	0.471	0.909	0.865	0.576	Not significant
**TFAP2_disc1**	3.644	0.000	10.000	0.900	0.691	0.850	0.021	Nominally significant (P<0.05)
**YY1_disc1**	2.745	0.000	13.000	0.615	0.792	0.805	0.581	Not significant
**YY1_known7**	5.327	0.000	7.000	0.714	0.476	0.530	0.453	Not significant
**YY2_1**	7.136	0.000	10.000	0.600	0.900	0.865	0.754	Not significant
**YY2_2**	3.508	0.000	7.000	0.571	0.872	1.064	1.000	Not significant
**ZBTB14_1**	3.722	0.000	27.000	0.259	0.271	0.338	0.019	Nominally significant (P<0.05)

**ZBTB14_3**	3.611	0.000	53.000	0.453	0.664	0.899	0.583	Not significant
**ZBTB6_1**	2.635	0.000	18.000	0.333	-0.086	-0.146	0.238	Not significant
**ZBTB7A_disc1**	3.466	0.000	38.000	0.316	0.206	0.322	0.034	Nominally significant (P<0.05)
**ZBTB7A_disc2**	4.717	0.000	20.000	0.400	0.286	0.430	0.503	Not significant
**ZNF143_known1**	3.194	0.000	23.000	0.304	0.233	0.162	0.035	Nominally significant (P<0.05)

Supplementary Table 5 | Known GWAS signals colocalizing with islets eQTLs.

Loci index	GWAS Locus Gene Name	GWAS Traits	GWAS SNP	Allele	eGene	Studies	InsPIRE	Details
**1**	ABO	Disposition index	rs505922	C	ABO	Fadista et al. (2014); van de Bunt, et al. (2015)	No evidence	COLOC < 0.2, no RTC
**2**	ADCY5	FGlu,T2D	rs11708067	A	ADCY5	Van de Bunt et al. (2015); Varshney, et al. (2017)	Replicated	
**3**	AMT	Fastin glucose	rs11715915	C/T	RBM6	Van de Bunt et al. (2015)	No evidence	Best COLOC = 0.36 (BSN-AS2); best RTC = 0.89 (CCDC51)
**4**	ANK1	T2D	rs515071	C	NKX6-3	Varshney, et al. (2017)	Not tested (R^2 < 0.5)	
		T2D	rs516946	C	NKX6-3	Varshney, et al. (2017)		
		Corrected insulin response	rs12549902	A/G	NKX6-3	Van de Bunt et al. (2015)	Replicated	
**5**	AP3S2	T2D	rs2028299	C	AP3S2	Varshney, et al. (2017)	Yes (other snp, R^2 = 0.897)	Same loci, different lead SNPs investigated
		T2D	rs2028299	A/C	AP3S2	Van de Bunt et al. (2015)		
		T2D	rs2028299	C/A	AP3S2	Fadista et al. (2014)		
**6**	CENTD2-ARAP1	FGlu,FGluBMIadj,Fproinsuli n	rs11603334	G	STARD10	Varshney, et al. (2017)	Replicated	
		Fasting glucose	rs11603334	G/A	STARD10	Van de Bunt et al. (2015)	Replicated	
		T2D	rs1552224	A/C	STARD10	Van de Bunt et al. (2015)	Yes (other snp, R^2 = 0.951)	Same loci, different lead SNPs investigated
		T2D	rs1552224	A	STARD10	Varshney, et al. (2017)		
**7**	VPS13C-C2CD4AB-NLF1	T2D	rs7172432	A	C2CD4B	Varshney, et al. (2017)	No evidence (R^2 = 1)	COLOC (rs8037894) = 0.67; no RTC
		FGlu	rs11071657	A	C2CD4B	Varshney, et al. (2017)	other snp (R^2 < 0.5)	COLOC (rs4502156 = 0.92, R^2=0.33); no eQTLs in the previously reported

								candidate
		2hGlu	rs17271305	G	VPS13C	Varshney, et al. (2017)	Not tested	No eQTL in VPS13C
**8**	CDC123/CAMK1D	T2D	rs11257655	T	CAMK1D	Varshney, et al. (2017)	Replicated	
		T2D	rs11257655	T/C	CAMK1D	Van de Bunt et al. (2015)		
		T2D	rs12779790	G	CAMK1D	Varshney, et al. (2017)	Not tested	Different lead SNP investigated. R^2 with rs11257655 = 0.82
		T2D	rs10906115	A	CAMK1D	Varshney, et al. (2017)		Different lead SNPs investigated. R^2 with rs11257655 = 0.19
**9**	DGKB/TMEM195	T2D; Fasting glucose	rs2191349	T/G	DGKB	Van de Bunt et al. (2015)	Replicated (other snp, R^2 = 0.979)	Same loci, different lead SNPs investigated
		T2D; Fasting glucose	rs17168486	T/C	DGKB	Van de Bunt et al. (2015)	Replicated	
**10**	DNLZ	FGlu	rs3829109	G	GPSM1	Varshney, et al. (2017)	No evidence	R^2 with rs11787792 = 0.67; best COLOC (INPP5E) = 0.47; RTC (SNAPC4) = 0.75
	GPSM1	T2D	rs11787792	A	GPSM1	Varshney, et al. (2017)	No (other snp, R2 < 0.5)	R^2 rs28505901 = 0.55; rs78403475 = 0.079; rs11793035 = 0.048
**11**	ERAP2	2hGlu	rs1019503	A	ERAP2	Varshney, et al. (2017)	Not tested	
	ERAP2	2-hour glucose	rs1019503	G/A	ERAP2	Fadista et al. (2014)		
**12**	FADS1	Fasting	rs174550	T/C	FADS1	Van de Bunt et al. (2015)	Replicated	
		FGlu	rs174550	C	FADS1	Varshney, et al. (2017)		
**13**	HIP1	FIns	rs1167800	A	PMS2P3	Varshney, et al. (2017)	Not tested	No eQTL in any of the candidate genes.
	HIP1	FIns	rs1167800	A	STAG3L1	Varshney, et al. (2017)		
	HIP1	FIns	rs1167800	A	GTF2IP1	Varshney, et al. (2017)		

**14**	HMG20A	T2D	rs7178572	A	HMG20A	Varshney, et al. (2017)	Replicated (other snp, R^2 = 0.908)	Same loci, different lead SNPs investigated
		T2D	rs7177055	A	HMG20A	Varshney, et al. (2017)	Replicated (other snp, R^2 = 1)	Same loci, different lead SNPs investigated
**15**	IGF2BP2	2hGluBMIadj,FGlu	rs7651090	G	IGF2BP2	Varshney, et al. (2017)	Replicated	
		T2D	rs4402960	T	IGF2BP2	Varshney, et al. (2017)	Replicated (other snp, R^2 = 1)	Same loci, different lead SNPs investigated
**16**	KCNK16	T2D	rs1535500	T	KCNK17	Varshney, et al. (2017)	Not tested	KCNK16 have no eQTLs. KCNK17 have two eQTLs (rs10947805, rs3807042)
		T2D	rs1535500	G/T	KCNK17	Fadista et al. (2014)		
**17**	KLHDC5	T2D	rs10842994	C	KLHL42	Varshney, et al. (2017)	Replicated (other snp, R^2 = 0.979)	Same loci, different lead SNPs investigated
**18**	MADD	Proinsulin	rs10501320	G	MADD	Varshney, et al. (2017)	Replicated	
		Fastin glucose	rs10501320	G/C	MADD	Van de Bunt et al. (2015)		
		Fastin glucose	rs10838687	T/G	ACP2	Van de Bunt et al. (2015)	Not tested	Different lead SNPs investigated. R^2 with rs10501320 = 0.08
		Fastin glucose	rs7944584	A/T	MADD	Van de Bunt et al. (2015)	only RTC	Different lead SNPs investigated. R^2 with rs10501320 = 0.929
**19**	MPHOSPH9	T2D	rs1727313	C	ABCB9	Varshney, et al. (2017)	Replicated (other snp, R^2 = 0.921)	Same loci, different lead SNPs investigated
**20**	MTNR1B	FGlu,T2D	rs10830963	G	MTNR1B	Varshney, et al. (2017)	Not tested	Gene not expressed enough for exon filters
		FGlu	rs10830962	C	MTNR1B	Varshney, et al. (2017)		
		T2D; Fasting glucose; HOMA-B; Corrected insulin reponse	rs10830963	G/C	MTNR1B	Van de Bunt et al. (2015)		

		T2D; Fasting glucose; HOMA-B; Corrected insulin reponse	rs10830963	C/G	MTNR1B	Fadista et al. (2014)		
**21**	PCSK1	Fasting glucose	rs4869272	T/C	CTD-2260A17.2	Van de Bunt et al. (2015)	Not tested	
**22**	SIX3 - SIX2	FGlu	rs895636	C	SIX3-AS1	Varshney, et al. (2017)	Replicated	
		FGlu	rs895636	C	AC012354.6	Varshney, et al. (2017)		
		FGlu	rs895636	C	SIX3	Varshney, et al. (2017)		
		FGlu	rs895636	C	RP11-89K21.1	Varshney, et al. (2017)		
**23**	TMEM163	T2D	rs6723108	T/G	MGAT5	Van de Bunt et al. (2015)	Not tested	eQTL in TMEM163 (rs6760237); eQTL in MGAT5 (rs6430497)
**24**	UBE2E2	T2D	rs1496653	A	UBE2E2	Varshney, et al. (2017)	Replicated (other snp, $R^2 = 1$)	Same loci, different lead SNPs investigated
**25**	WARS	Fastin glucose	rs3783347	G/T	WARS	Van de Bunt et al. (2015)	Replicated	
**26**	ZFAND6	T2D	rs11634397	G/A	LINC00927	Van de Bunt et al. (2015)	Not tested	No eQTLs in any candidate gene
**27**	ZMIZ1	T2D	rs12571751	A/G	ZMIZ1	Van de Bunt et al. (2015)	No evidence	COLOC < 0.04; no RTC

Supplementary Table 6 | Oligonucleotide primers used for the transcriptional reporter assays.

Primer	Sequence (5’-3’)	Amplified region (hg19)
Haplotype A_Forward<br>Haplotype A_Reverse	GAGTTGCTGGAATGGAGGAG<br>CATGCGTCGCAATGTCTTT	chr7:15,055,310-15,056,074
Haplotype B_Forward<br>Haplotype B_Reverse	TGCTAAGGCTAACCATAAACCA<br>TCAGCCAGCTGAAAGCTAGA	chr7:15,064,030-15,064,621

Supplementary Table 7 | Oligonucleotide probes used for the electrophoretic mobility shift assays.

Variant	Sequence (5’-3’) ‡	Position
rs10228796	ACTGGGTT(**C/G)**TACCTTAA	chr7:15064182-15064198
rs10258074	TGGCAACC**(A/T)**GTCTCCTA	chr7:15064208-15064224
rs2191348	TTACCAAT**(G/T)**CAATACCA	chr7:15064247-15064263
rs2191349	AGGATCAT(**G/T)**TAAGACCA	chr7:15064301-15064317

‡Variant alleles are in bold.

Supplementary Discussion

Influence of sample size in eQTL discovery and comparison with GTEx eQTLs.

Sample size influences eQTL discoveries, with the current knowledge indicating that larger eQTLs studies are able to find more tissue or cell specific eQTLs (Aguet, et al. 2017). Therefore, with larger samples sizes a higher absolute number of eQTLs are expected to be shared, but the percentage of shared signals is expected to be smaller. To test the influence of sample size on the discovery of shared genetic signals between our islet sample and GTEx eQTLs, we randomly selected 149 islets samples (the size of the GTEx pancreas study, performed an islet eQTL analysis and then estimated the signals shared with GTEX tissues (up to 382 samples). In the figure bellow, we observe that multiple tissues capture the same eQTLs signals as islets (N = 149), including adipose (0.89), LCLs (0.84), esophagus (0.84), and pancreas (0.84) which has the smallest sample size of the four tissues. Overall, 0.53 to 0.89 of the 149 islet samples signals were shared with GTEx tissues compared to 0.4 to 0.73 of the 420 islet samples. This supports the current expectation that larger sample size eQTLs studies will identify a larger proportion of tissue or cell specific signals.

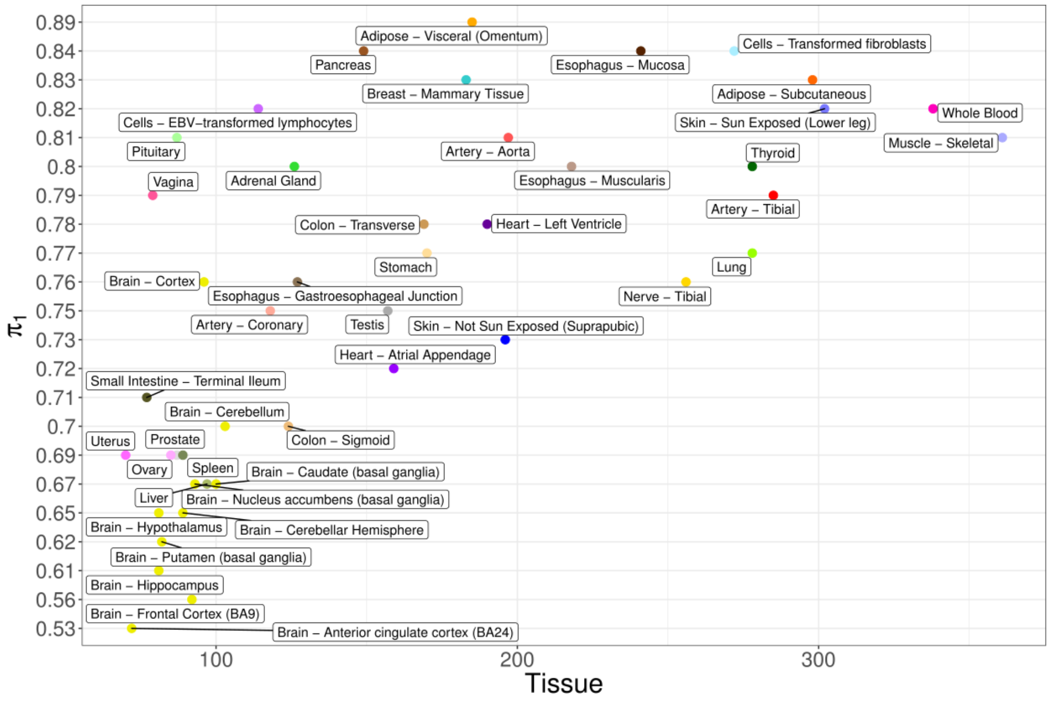

Exocrine contamination

To evaluate whether some of the eQTLs attributed to pancreatic islets may reflect exocrine pancreatic contamination, we compared the sets of eQTLs identified in the InsPIRE islet samples with the highest and lowest proportions of exocrine contamination (n=100 for each) and 100 randomly-selected GTEx whole pancreas samples. The estimated signal overlap between whole pancreas and islet eQTLs was greater in islet samples with the highest exocrine contamination than with the lowest exocrine contamination (π_1 75% vs 64%), indicating that some cell-specific effects were preserved even controlling for 25 PCs for the eQTL analysis (See Figure).

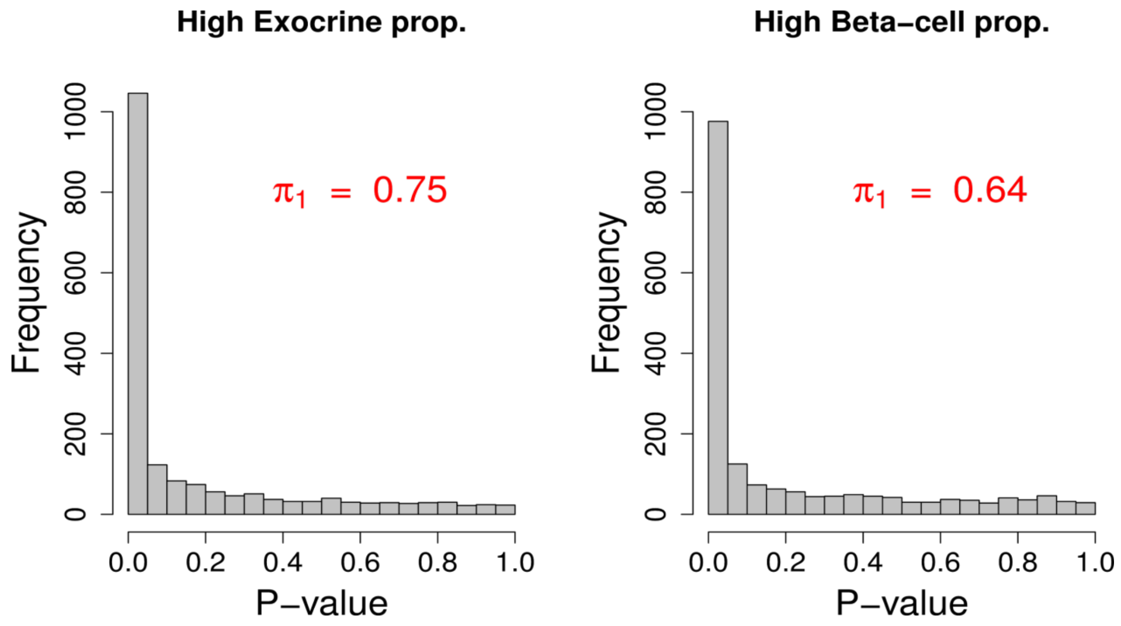

Supplementary Figures

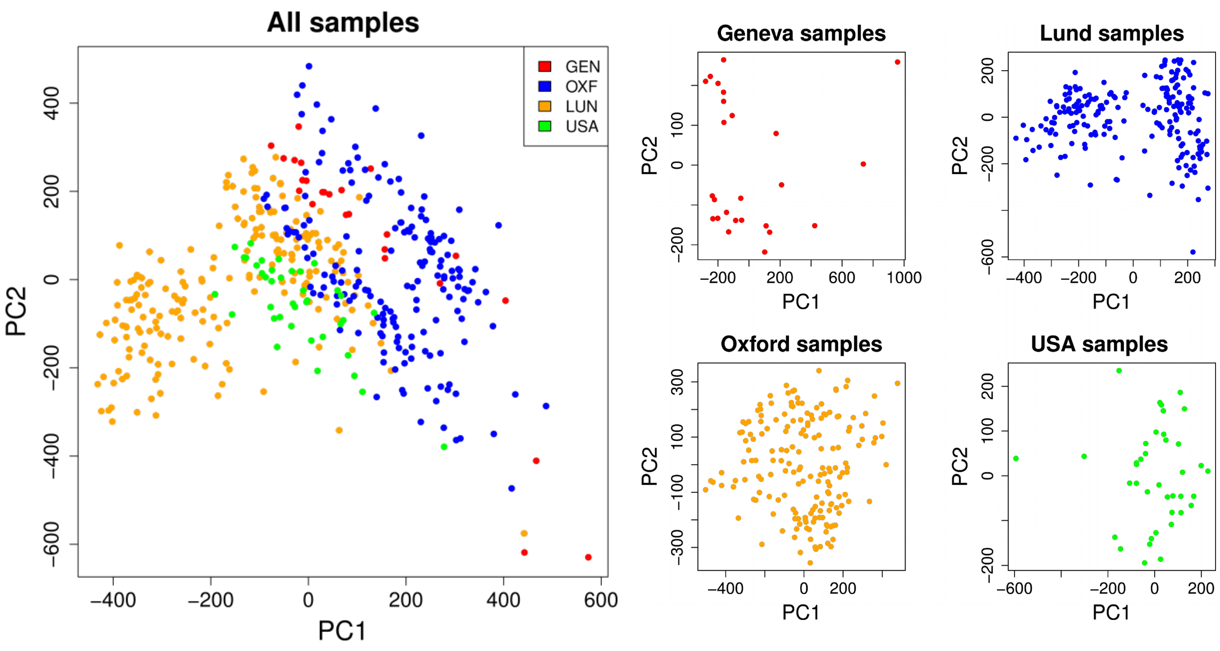

Supplementary Figure 1 | Principal component analysis (PCA) of islets samples. We calculate PCAs from the exon expression profiles per sample included in the InsPIRE project. Samples were re-quantified and normalized together to account for differences in the data production. The samples showed in the PCA analysis the differences due to experimental processing differences, with internal batch effects in the cases of samples from Oxford/Edmonton and Lund.

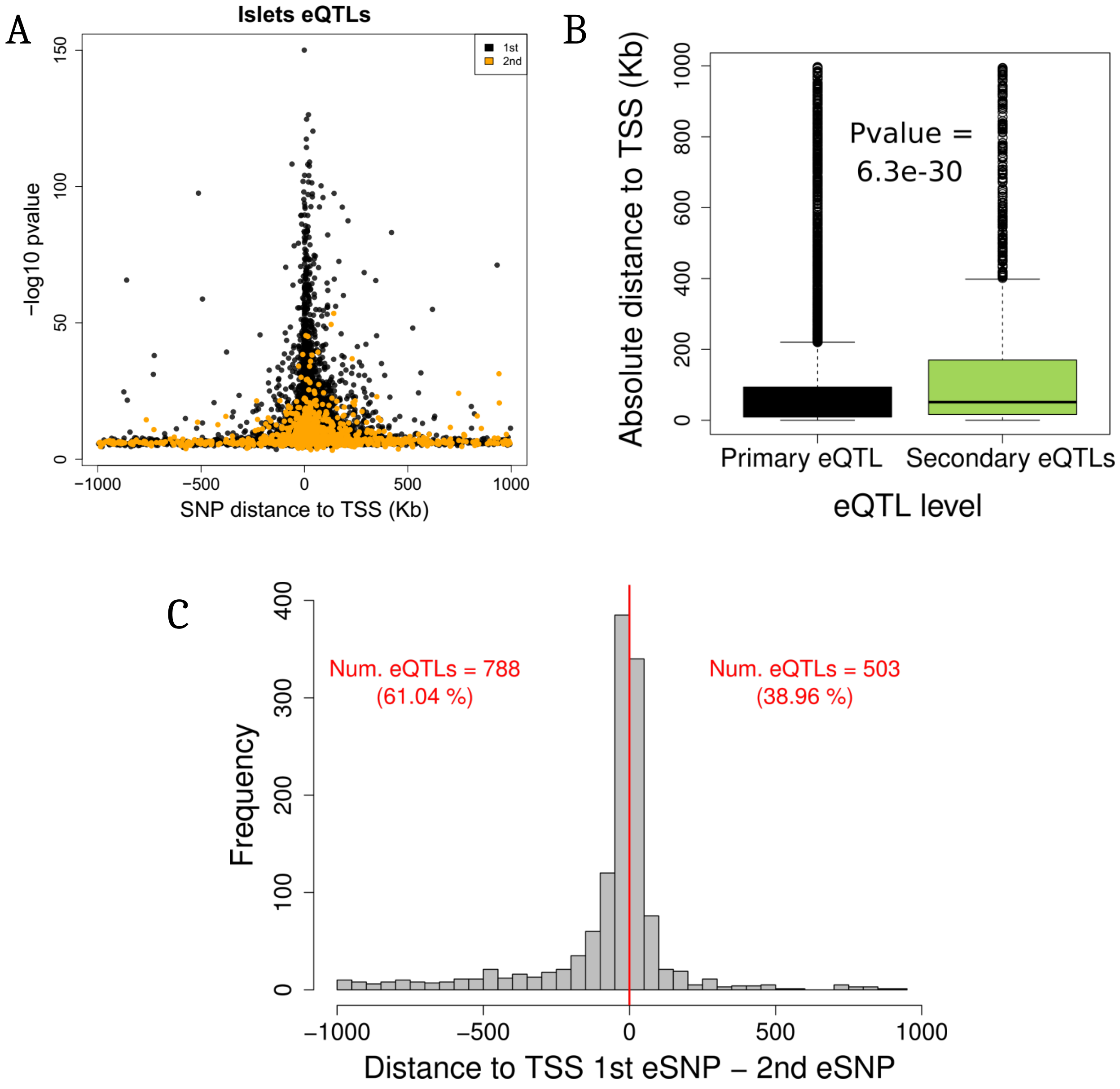

Supplementary Figure 2 | Properties of islets eQTL. A) –log10 p-value distribution of the lead eSNP per gene around the transcription start site (TSS) of the genes in black. Yellow values show the secondary signals discovered after conditional analysis. Both the primary and secondary sSNPs show smaller p-values around the TSS, however, the secondary signals are significantly further away from the TSS (**B**). Distance to TSS has been corrected by strand. **C)** Distance of the eSNPs around the TSS for those genes with 2 independent eQTLs (n = 1,290). The difference in the Kb distance between primary SNP (1st) and secondary SNP (2nd SNP as the highest variance explained in expression) independent eSNPs significantly affecting the expression of the same gene is expressed in negative values (left) if the primary eSNPs is closer to the TSS than the secondary eSNPs (N = 788). Positive values identify those eGenes in which the secondary eSNP is closer to the TSS than the primary (N= 503).

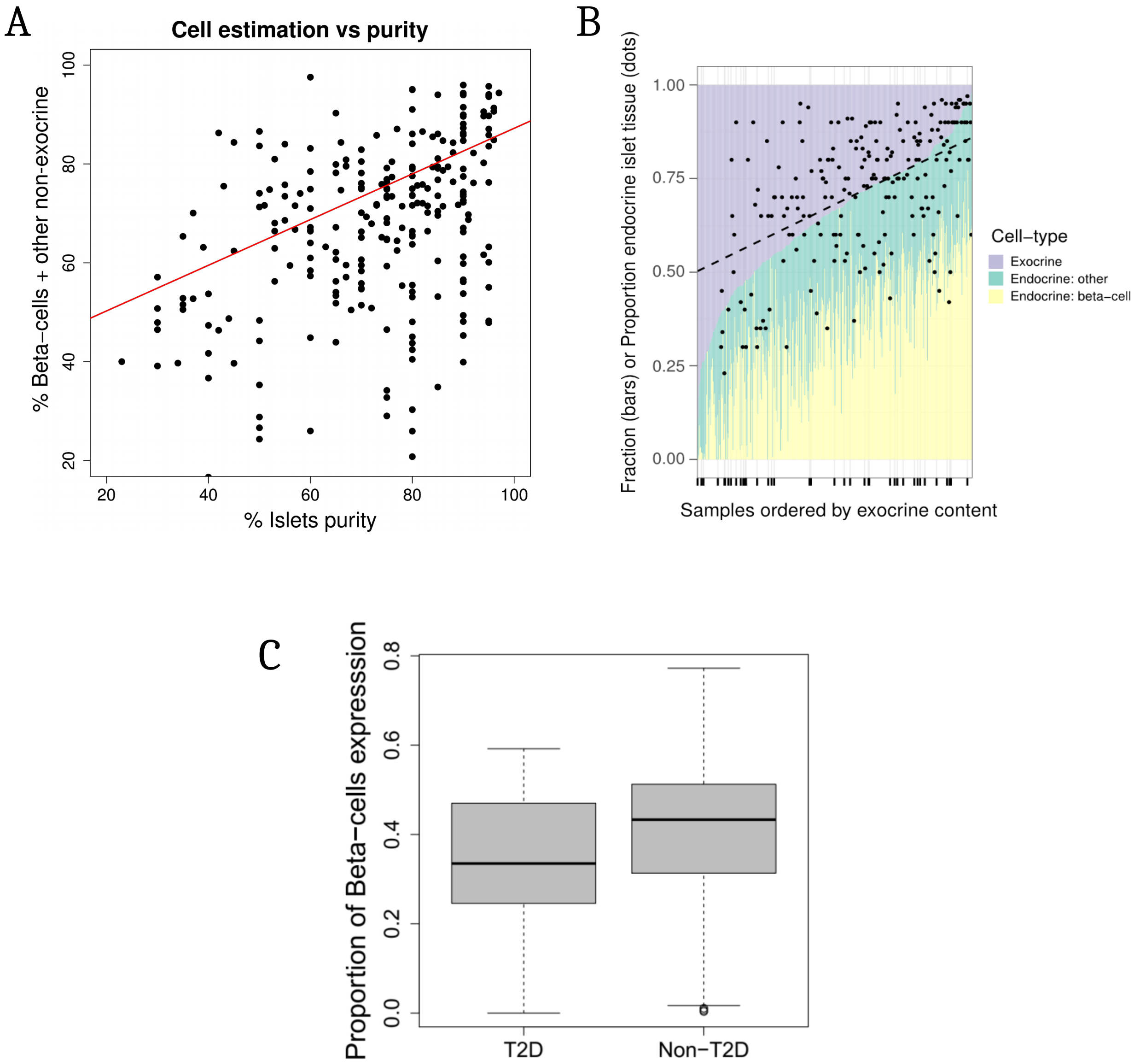

Supplementary Figure 3 | Cell deconvolution analysis. A) Percentage of purity for islets as measured in dithizone staining of the 253 samples compare to the estimated proportion of (beta-cells + other non-exocrine cell)/ total cell content in islets. The correlation between measured values of purity was ρ=-0.406 (p-value=1.4x10^{-11}). **B)** Estimates of the different types of cell considered in the 420 islets samples processed. The beta-cells proportion composition form per sample corresponded to a median of 58.8%, and 41.2% for non-beta-cell fractions. **C)** Percentage of Beta-cells expression detected in islets samples from individuals identified as diabetics (T2D), compare to non-T2D individuals.

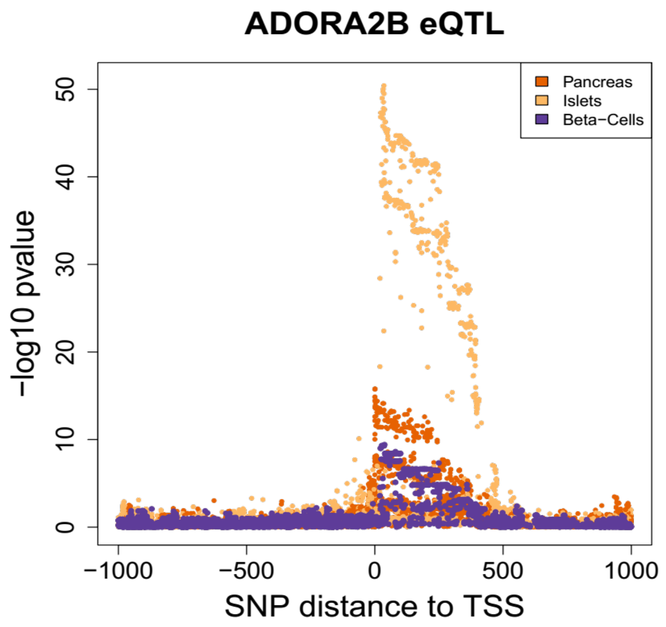

Supplementary Figure 4 | eQTL for ADORA2B in islets, beta-cells and pancreas samples. Each dot represent a SNPs in the cis window of ADORA2B and their distance in kb to the TSS. The y-axis shows the -log10 of the p-value for the association between a given SNP and the expression of the same exon in ADORA2B (exon ID =ENSG00000170425.3_15877177_15879060). For all tissues, at least one SNP was significant after multiple testing (FDR = 5%).

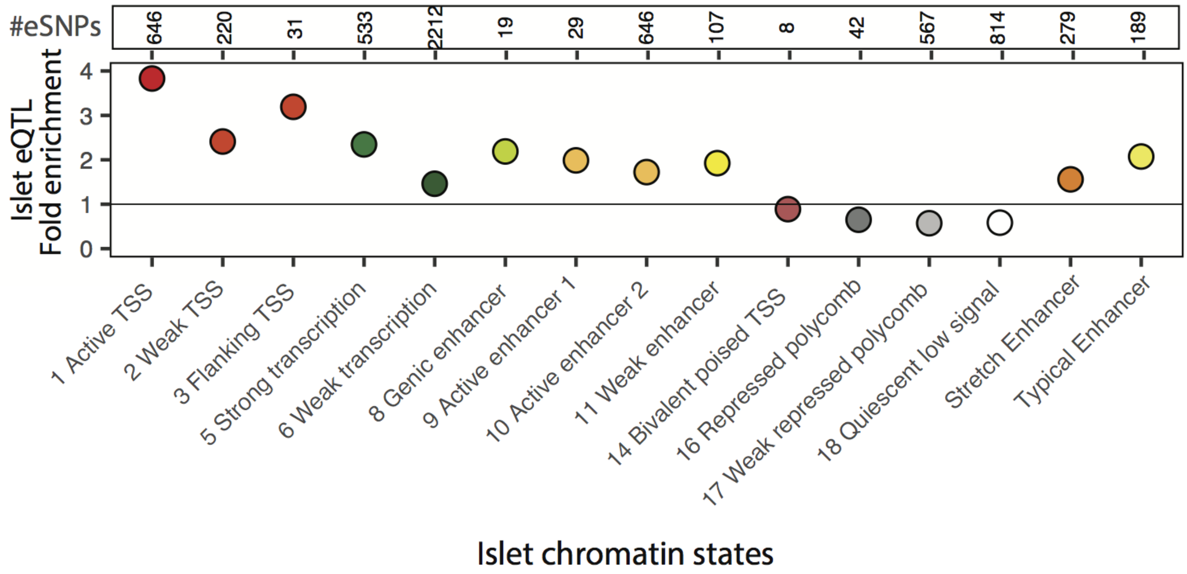

Supplementary Figure 5 | Islet eSNP overlap and enrichment in islet chromatin states. Top: Number of islet eSNPs (MAF>=0.2) in 13 islet chromatin states and stretch and typical enhancers. Bottom: Fold enrichment of islet eSNPs in chromatin states calculated using GREGOR [1]

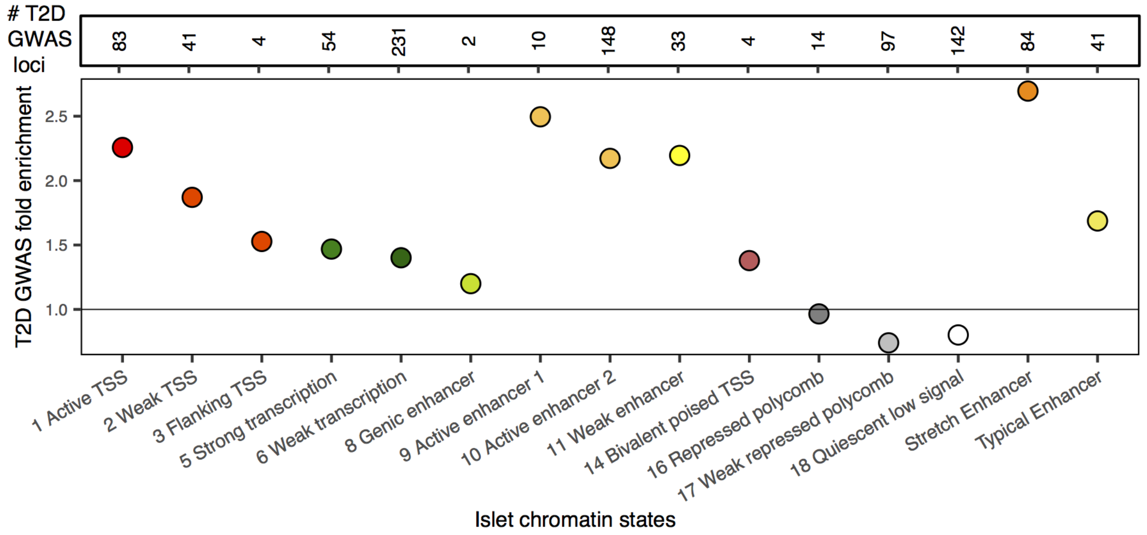

Supplementary Figure 6 | T2D GWAS overlap and enrichment in islet chromatin states. Top: Number of T2D GWAS loci occurring in each of the 13 islet chromatin states along with stretch and typical enhancers. Bottom: Fold enrichment of T2D GWAS in chromatin states calculated using GREGOR[1]

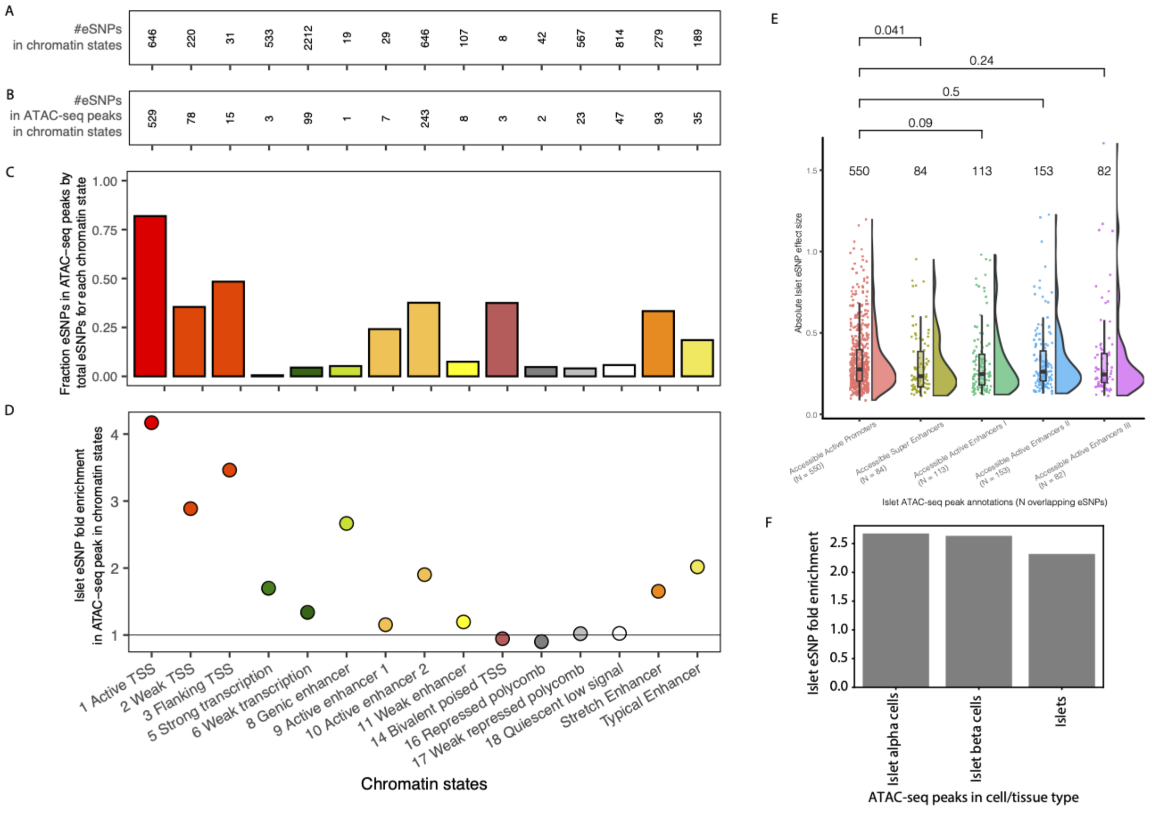

Supplementary Figure 7 | Islet eSNP enrichment in ATAC-seq peaks . A) Number of islet eSNPs overlapping with islet chromatin states and stretch/typical enhancers. **B)** Number of islet eSNPs in islet ATAC-seq peaks in chromatin states. **C)** Fraction of islet eSNPs in ATAC-seq peaks in each chromatin states. An eSNP overlap is considered if the eQTL lead eSNP or proxy SNP (LD r2>0.99) overlaps the feature. **D)** Fold enrichment of islet eSNPs to overlap ATAC-seq peaks within chromatin states. **E)** Absolute effect sizes for eSNPs occurring in islet ATAC-seq features such as active promoters, active enhancers I, II and III, and super enhancer segments overlapping 'robust' ATAC-seq peaks obtained from Miguel-Escalada et al. 2019. The number of eQTL overlaps in each annotation are shown in parentheses. P values are from Wilcoxon rank sum tests. **F)** Islet eSNP enrichment in ATAC-seq peaks from islets and islet alpha and beta cell clusters from the sci-ATAC-seq data from Rai et al. 2020.

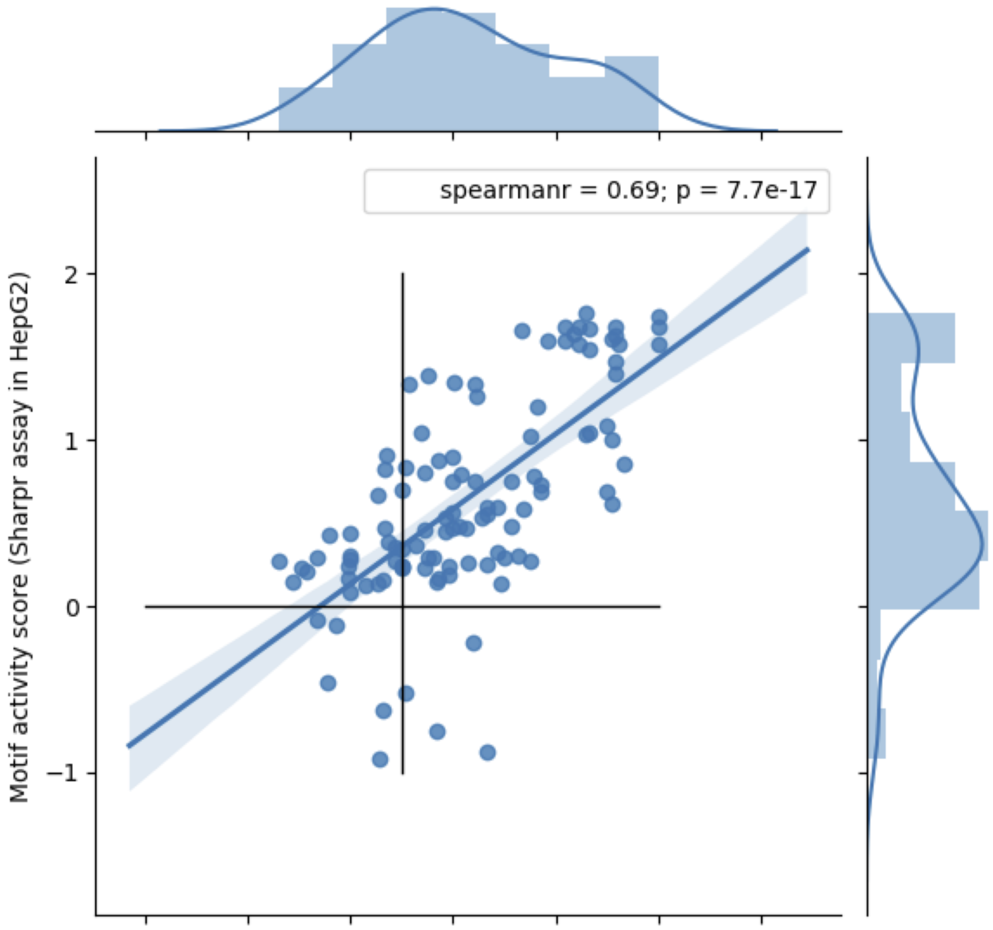

Supplementary Figure 8 | Comparison of TF motif directionality fractions with MPRA activity scores. Transcription factor motif activity scores from Sharpr MPRA in HepG2 vs Motif directionality fractions from islet eQTL and ATAC-seq TF footprinting data. TF Motifs that were reported to be either activating or repressive (P<0.01) from the MPRAs in both HepG2 and K562 are shown.

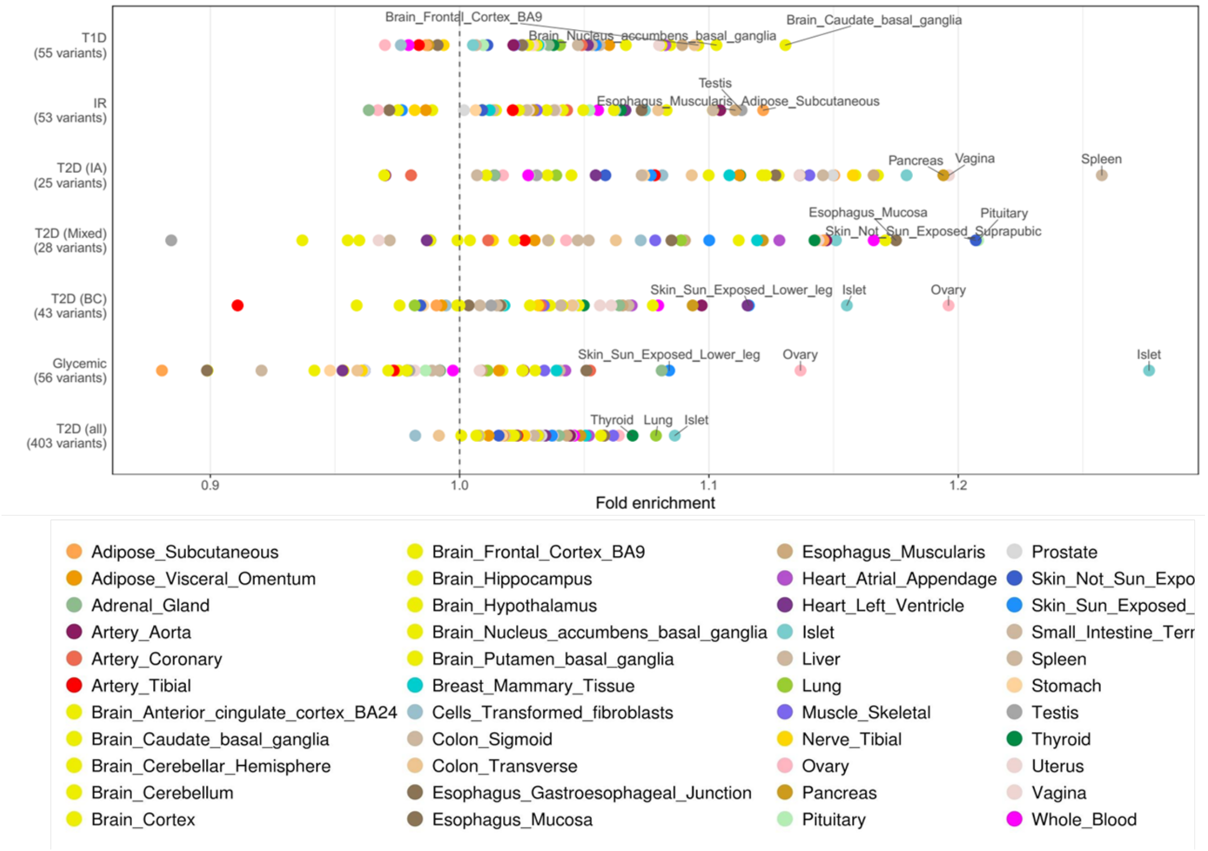

Supplementary Figure 9 | Tissue enrichment analysis in all GTEx tissues. Results support the conclusion show in the main figure that islets outperform other tissues for GWAS loci enrichment.

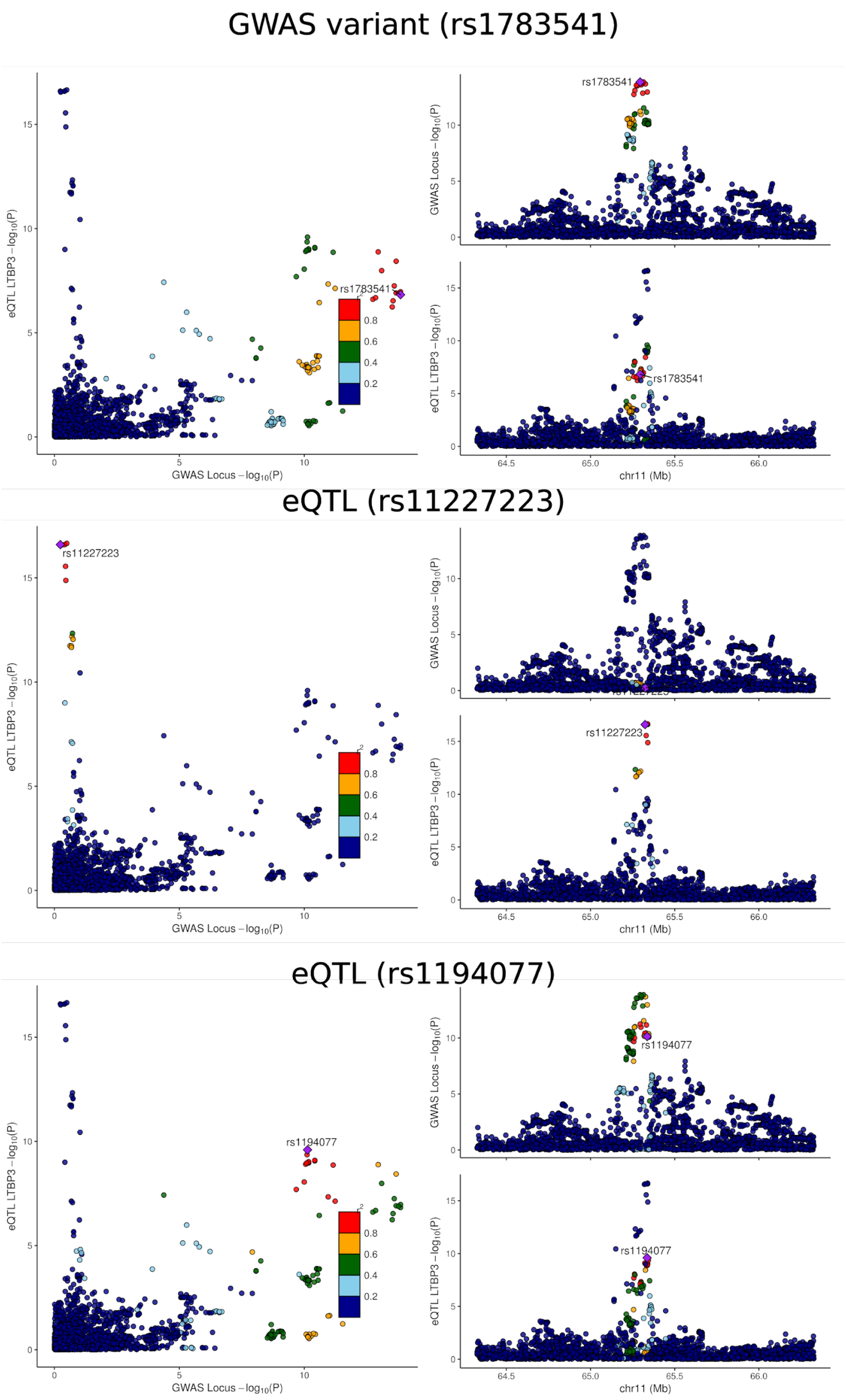

Supplementary Figure 10 | LocusCompare plot of the eQTL for the *MAP3K11* locus. Three LocusCompare are shown: i) Highlighting the T2D signal (rs1783541), then highlighting lead eSNP for the two eQTLs for *LTBP3*. The signal at rs1194077 appears coincident to the GWAS as reported by RTC (RTC score = 0.96), while COLOC report no colocalization with any variant.

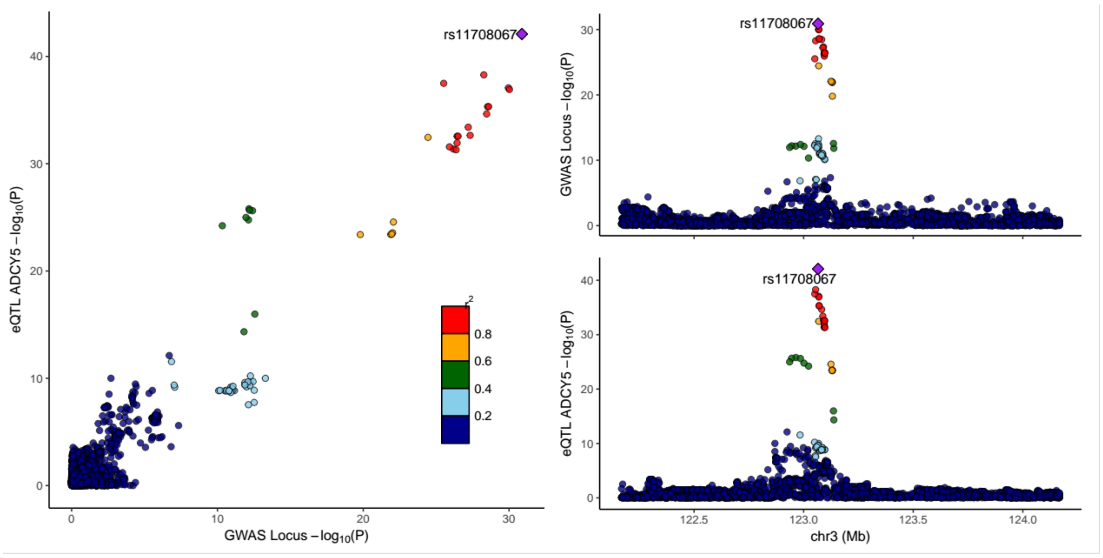

Supplementary Figure 11.1 | LocusCompare for the eQTL in *ACDY5* and GWAS loci known as *ACDY5*.

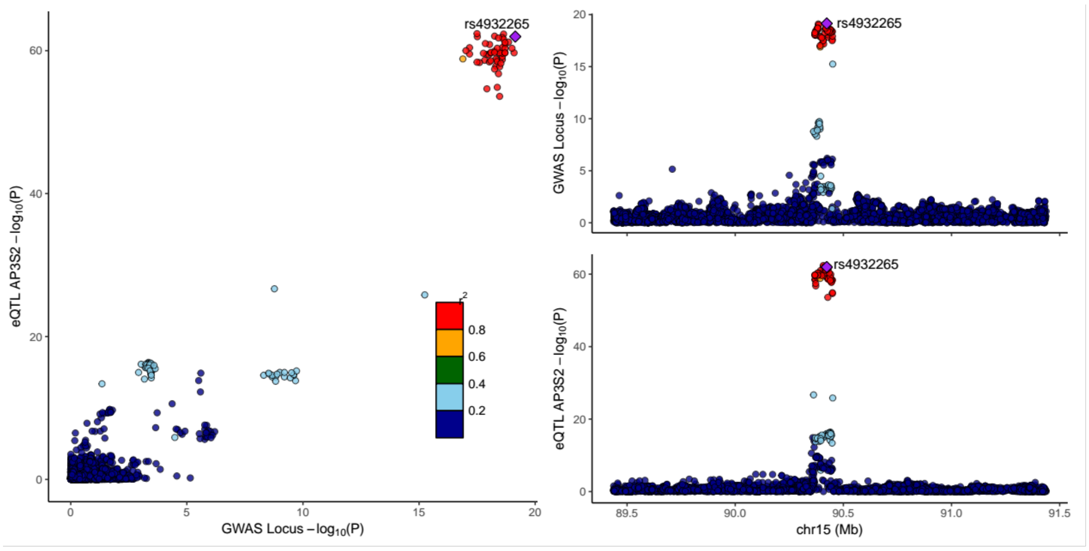

Supplementary Figure 11.2 | LocusCompara for the eQTL in *AP3S2* and GWAS loci known as *AP3S2*.

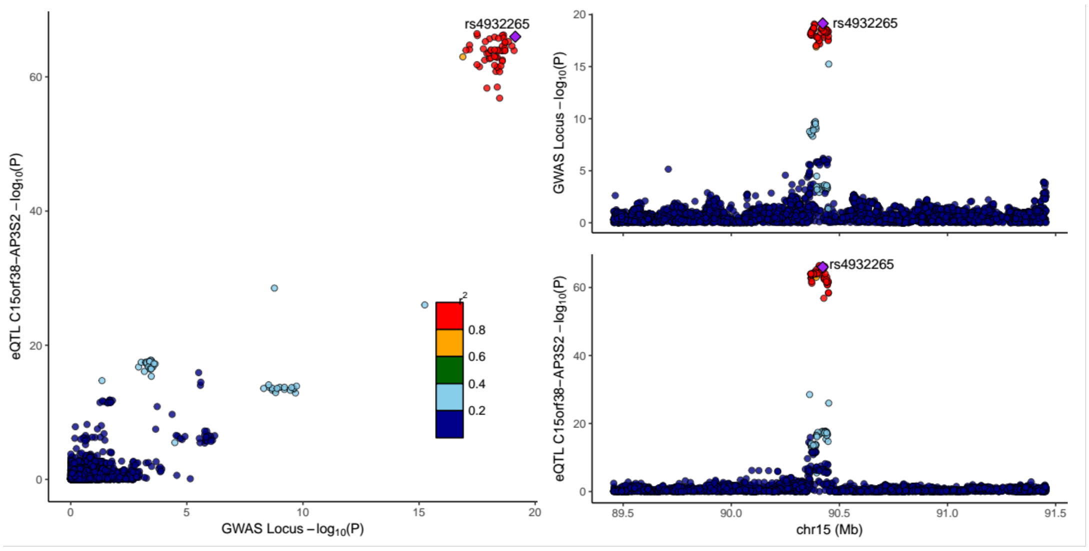

Supplementary Figure 11.3 | LocusCompare for the eQTL in *C15orf38-AP3S2* and GWAS loci known as *AP3S2*.

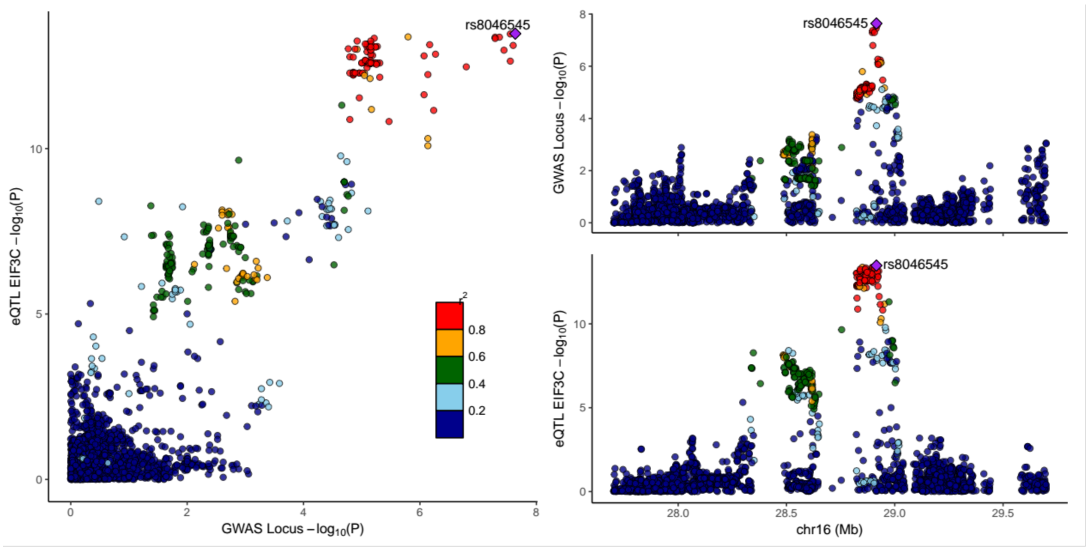

Supplementary Figure 11.4 | LocusCompare for the eQTL in *EIF3C* and GWAS loci known as *ATP2A1*.

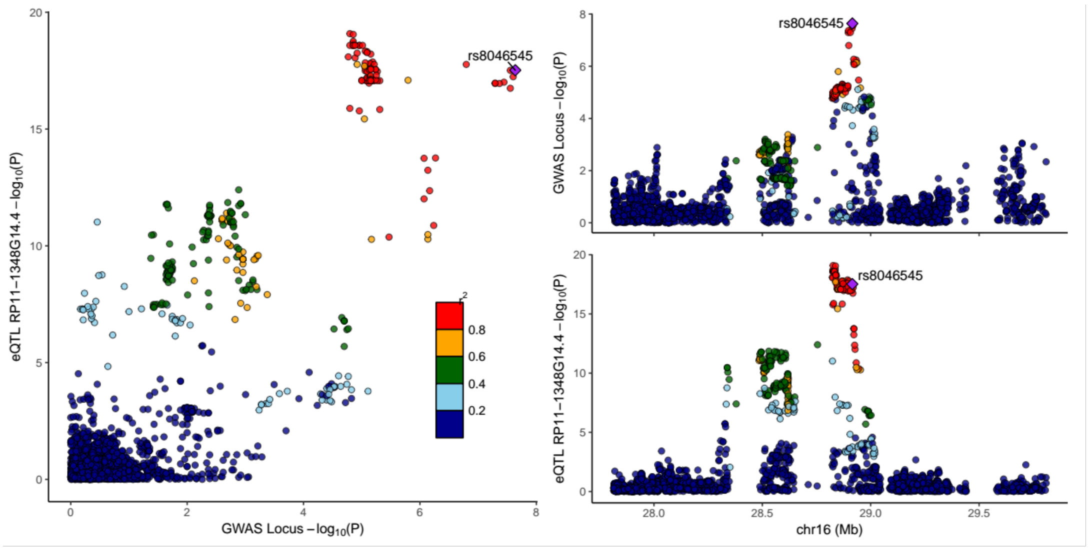

Supplementary Figure 11.5 | LocusCompare for the eQTL in *RP11-1348G14.4* and GWAS loci known as *ATP2A1*.

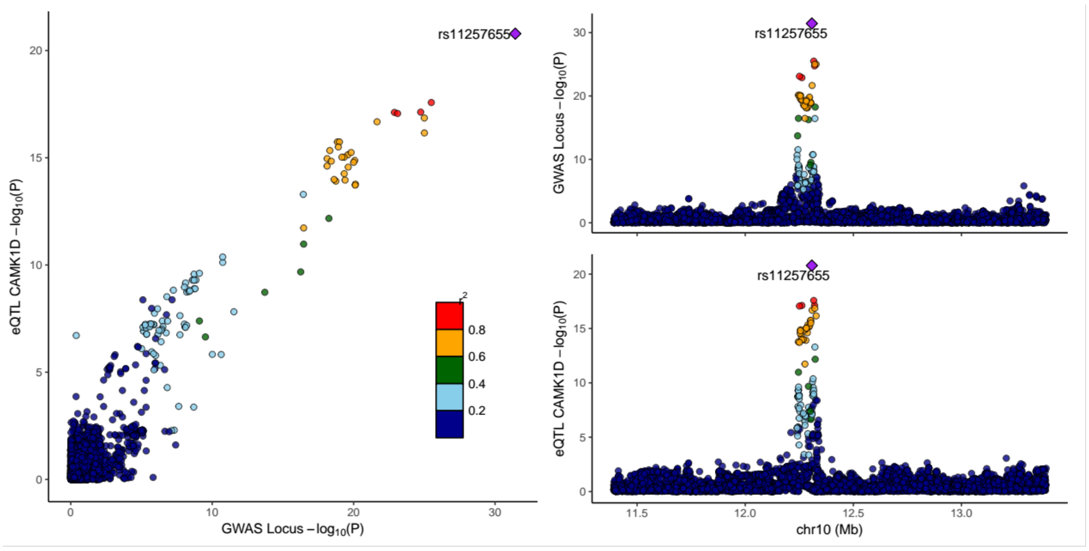

Supplementary Figure 11.6| LocusCompare for the eQTL in *CAMK1D* and GWAS loci known as *CDC123/CAMK1D*.

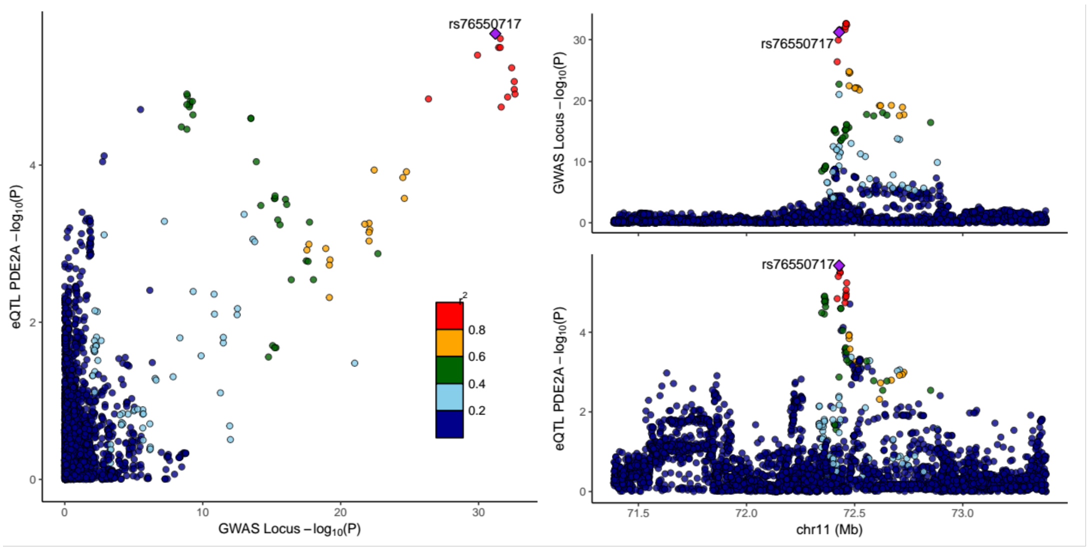

Supplementary Figure 11.7 | LocusCompare for the eQTL in *PDE2A* and GWAS loci known as *CENTD2-ARAP1*.

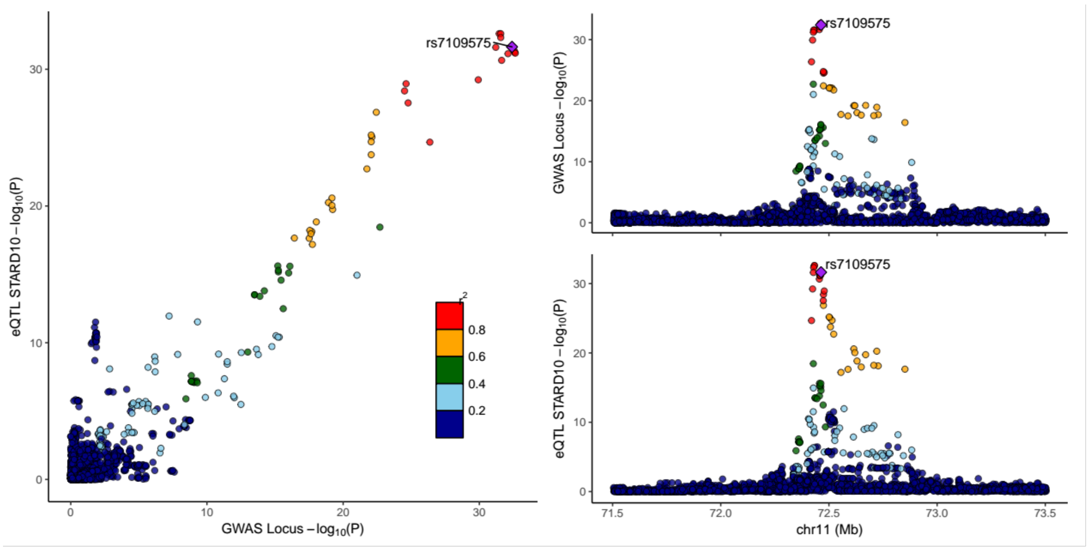

Supplementary Figure 11.8 | LocusCompare for the eQTL in STARD10 and GWAS loci known as *CENTD2-ARAP1*.

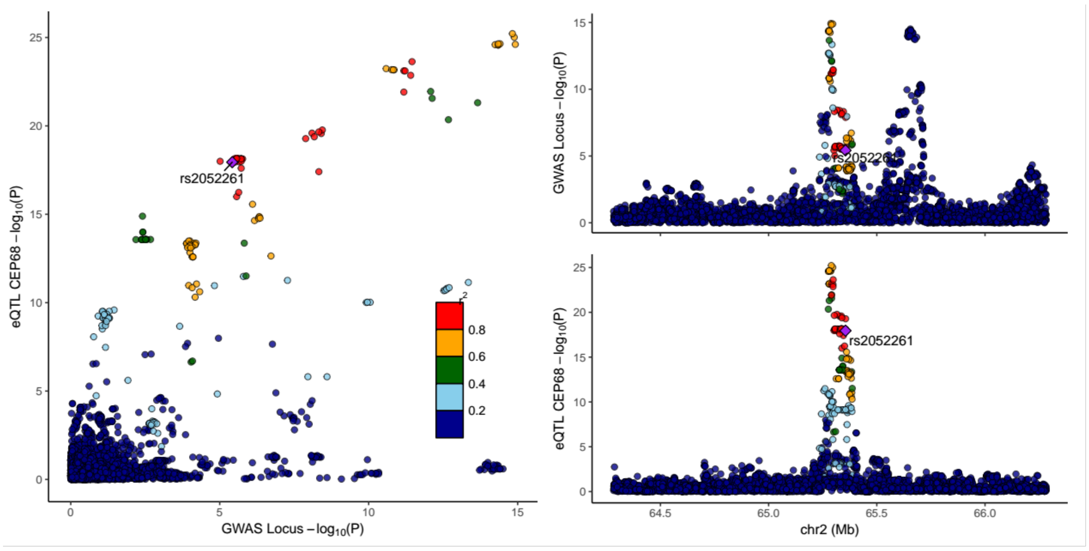

Supplementary Figure 11.9 | LocusCompare for the eQTL in *CEP68* and a secondary GWAS loci in *CEP68*.

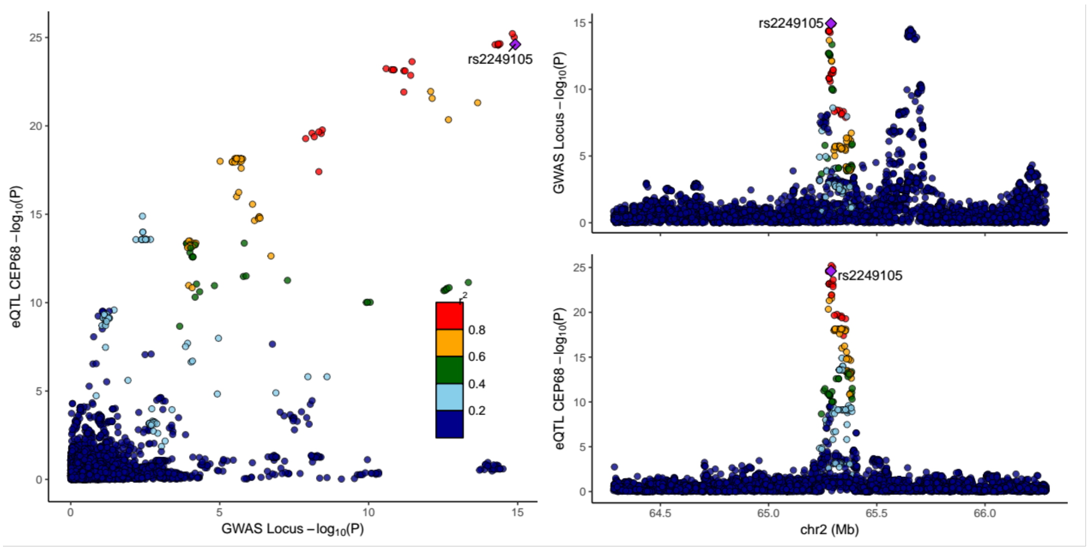

Supplementary Figure 11.10 | LocusCompare for the eQTL in *CEP68* and a primary GWAS loci in *CEP68*.

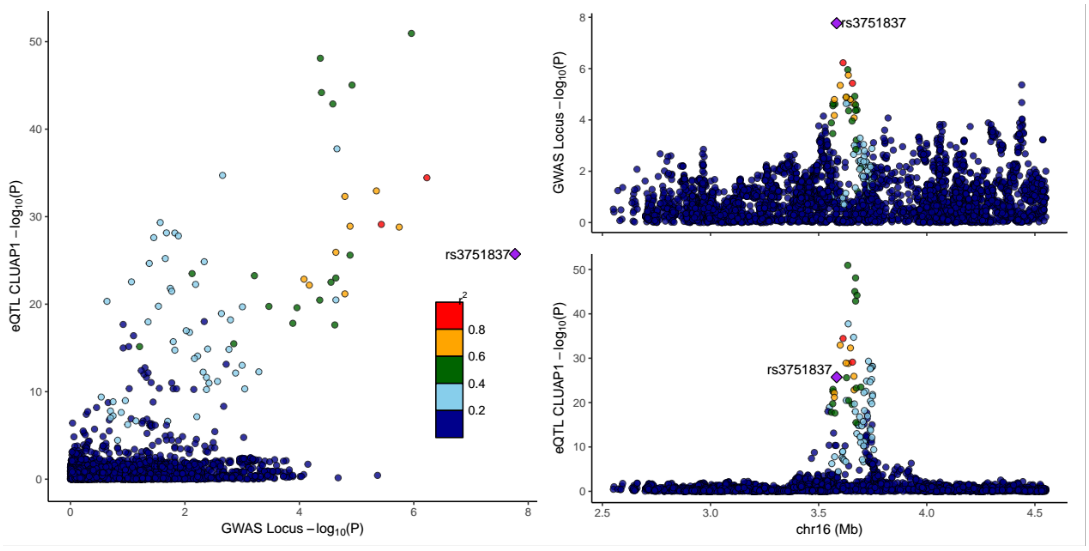

Supplementary Figure 11.11 | LocusCompare for the eQTL in *CLUAP1* and GWAS loci known as *CLUAP1*.

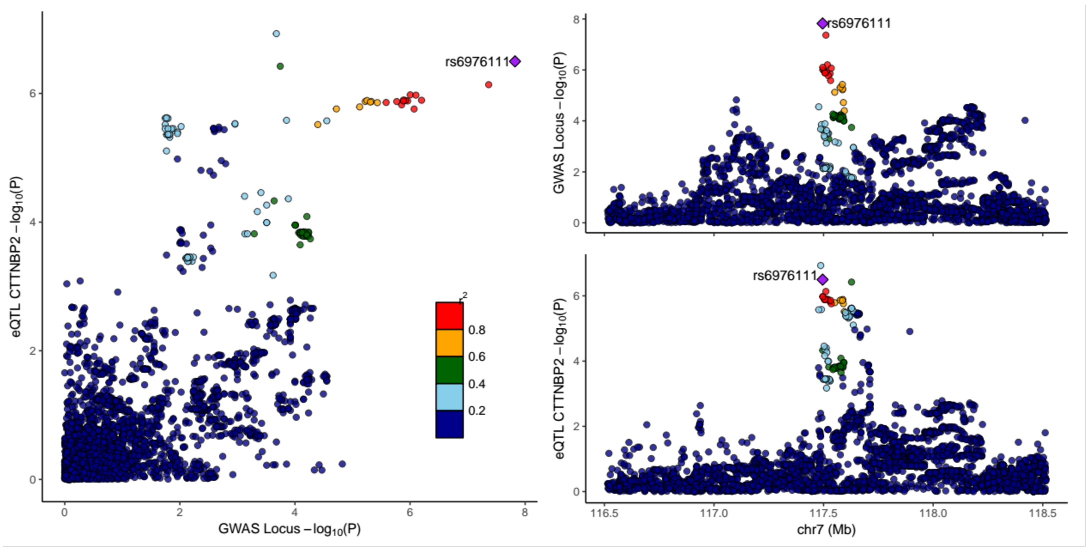

Supplementary Figure 11.12 | LocusCompare for the eQTL in *CTTNBP2* and GWAS loci known as *CTTNBP2*.

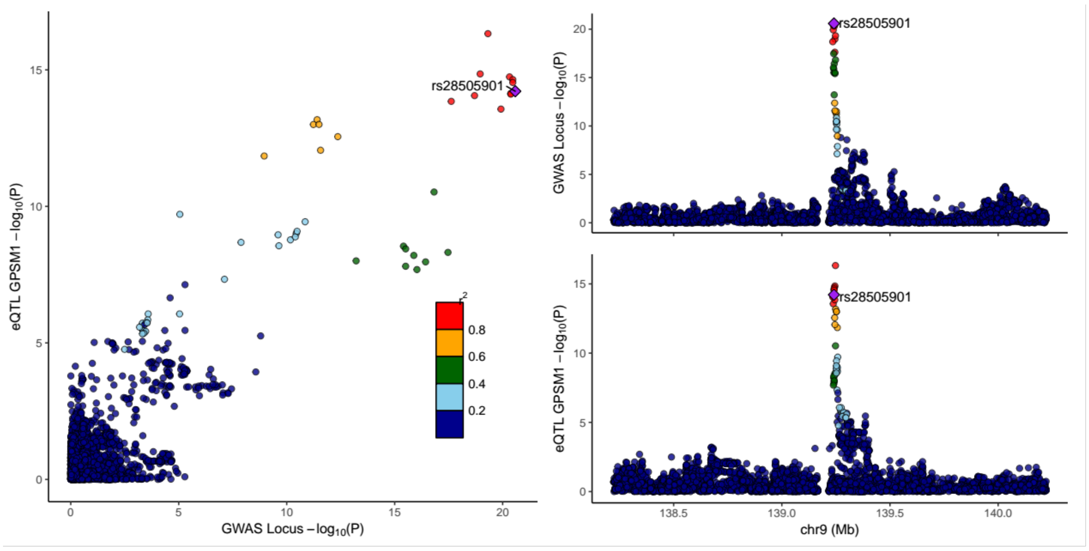

Supplementary Figure 11.13 | LocusCompare for the eQTL in *GPSM1* and GWAS loci known as *GPSM1*.

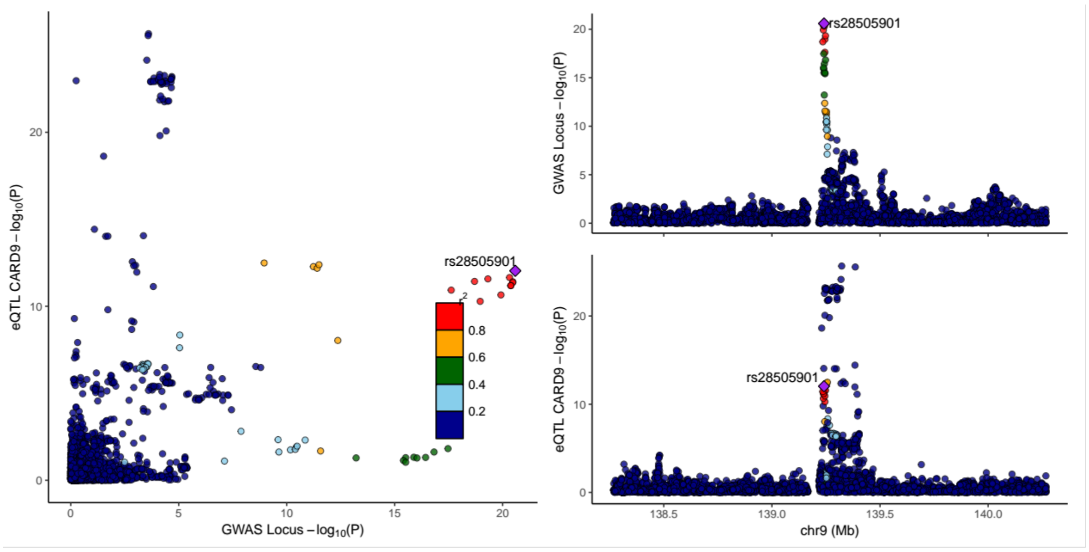

Supplementary Figure 11.14 | LocusCompare for the eQTL in *CARD9* and GWAS loci known as *GPSM1*.

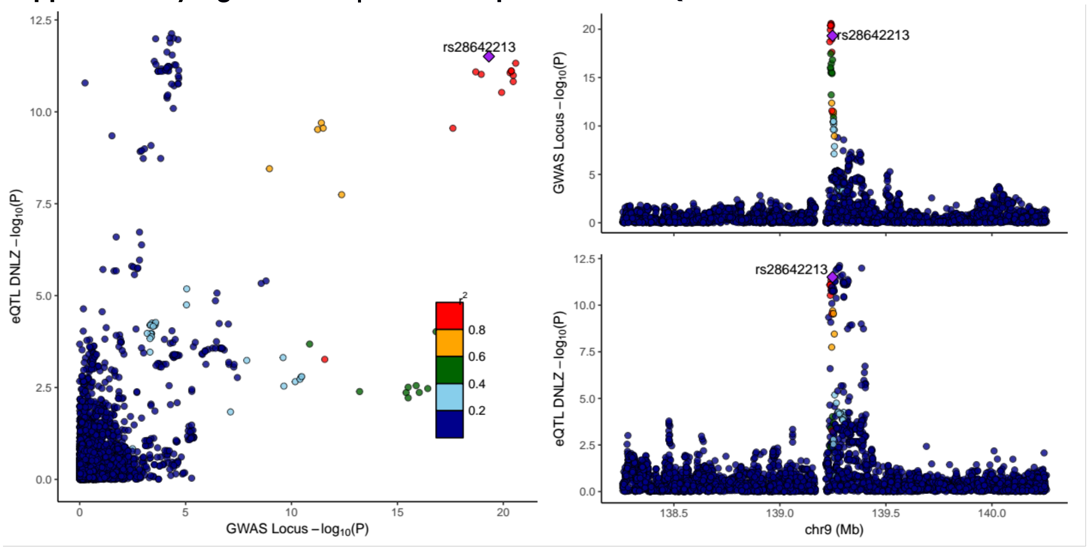

Supplementary Figure 11.15 | LocusCompare for the eQTL in *DNLZ* and GWAS loci known as *GPSM1*.

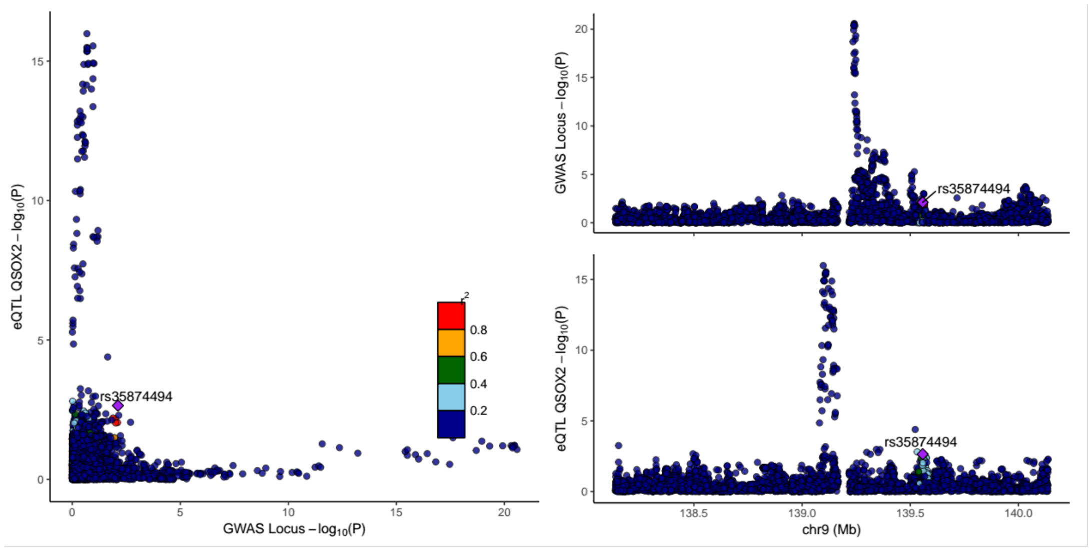

Supplementary Figure 11.16 | LocusCompare for the eQTL in *QSOX2* and GWAS loci known as *GPSM1*.

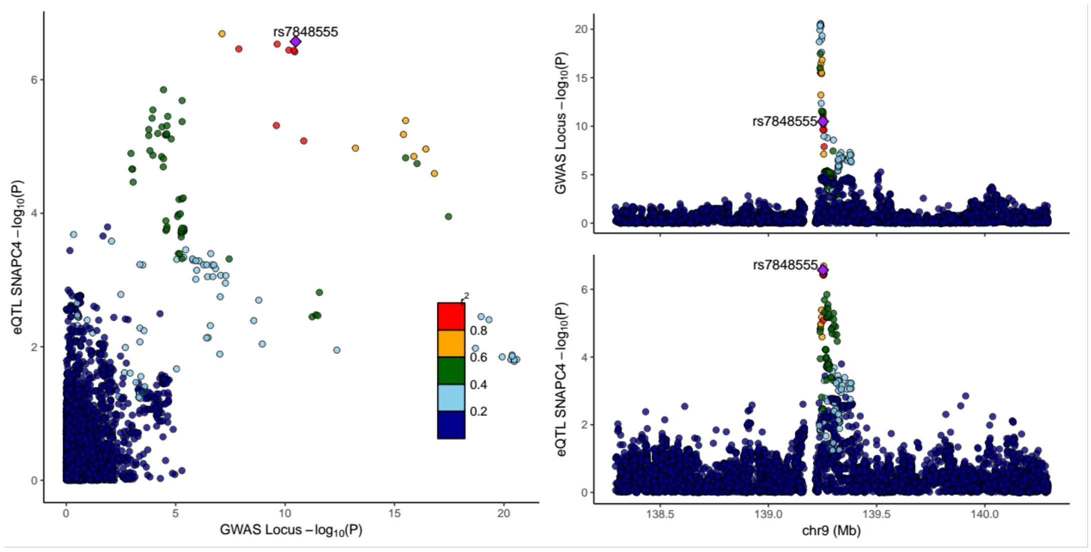

Supplementary Figure 11.17 | LocusCompare for the eQTL in *SNAPC4* and GWAS loci known as *GPSM1*.

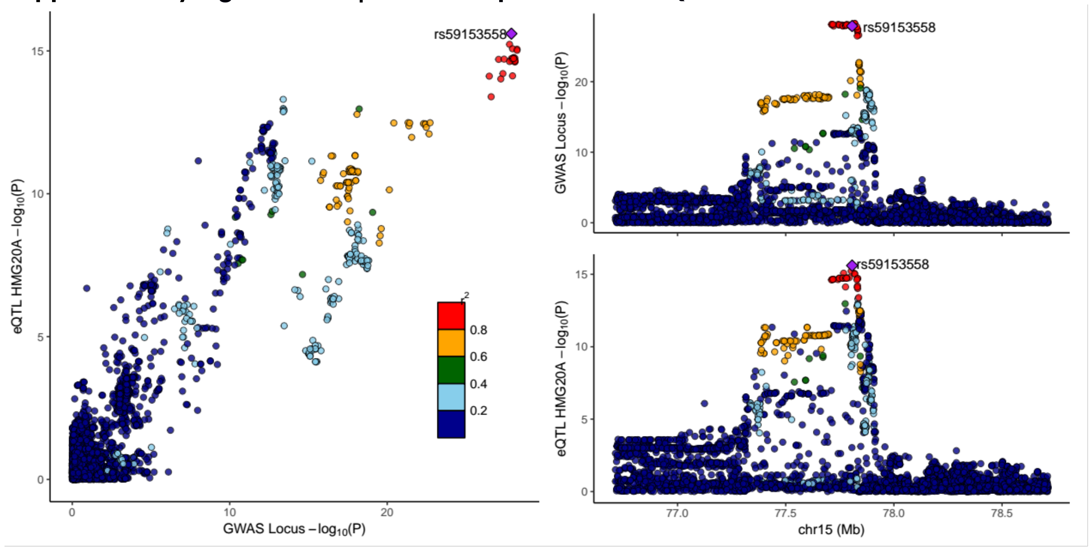

Supplementary Figure 11.18 | LocusCompare for the eQTL in *HMG20A* and GWAS loci known as *HMG20A*.

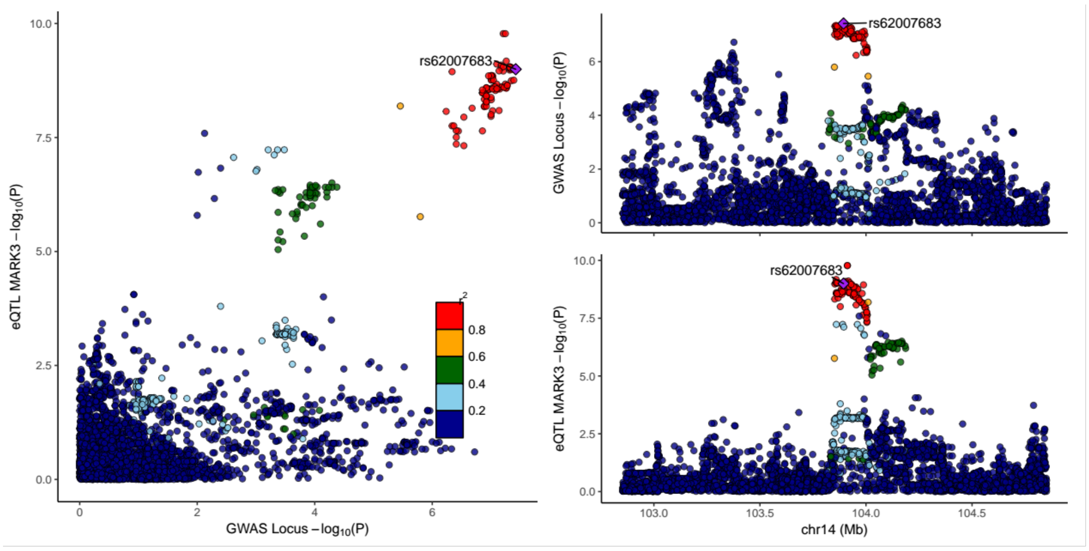

Supplementary Figure 11.19 | LocusCompare for the eQTL in *MARK3* and GWAS loci known as *MARK3*.

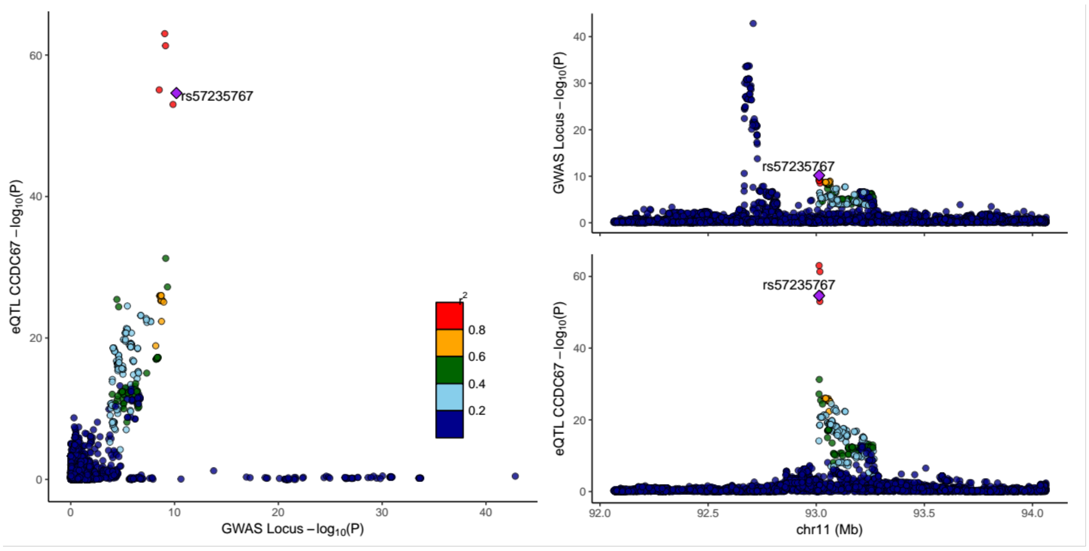

Supplementary Figure 11.20 | LocusCompare for the eQTL in *CCDC67* and a secondary GWAS loci in *MTNR1B*.

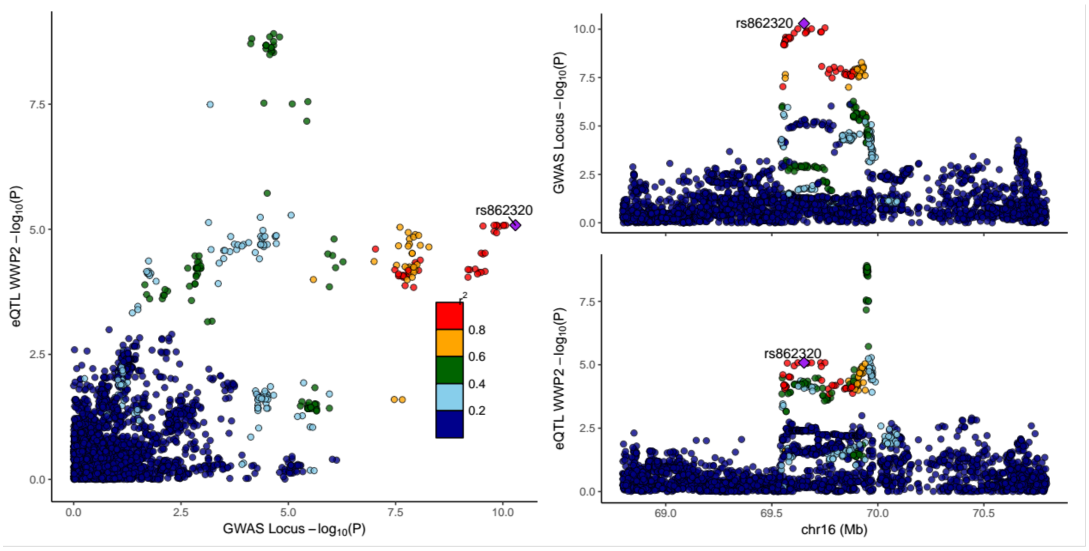

Supplementary Figure 11.21 | LocusCompare for the eQTL in *WWP2* and GWAS loci known as *NFAT5*.

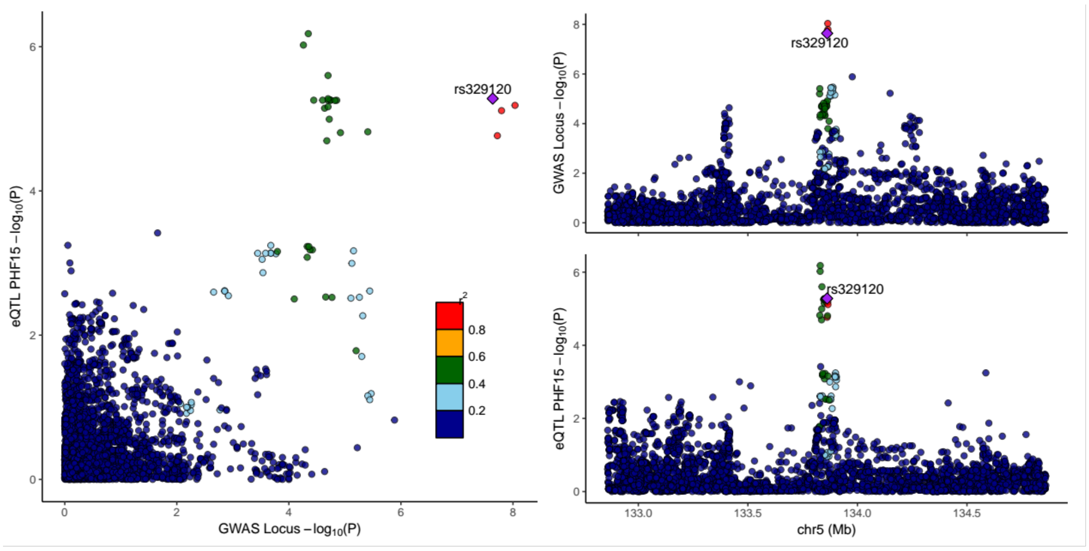

Supplementary Figure 11.22 | LocusCompare for the eQTL in *PHF15* and GWAS loci known as *PHF15*.

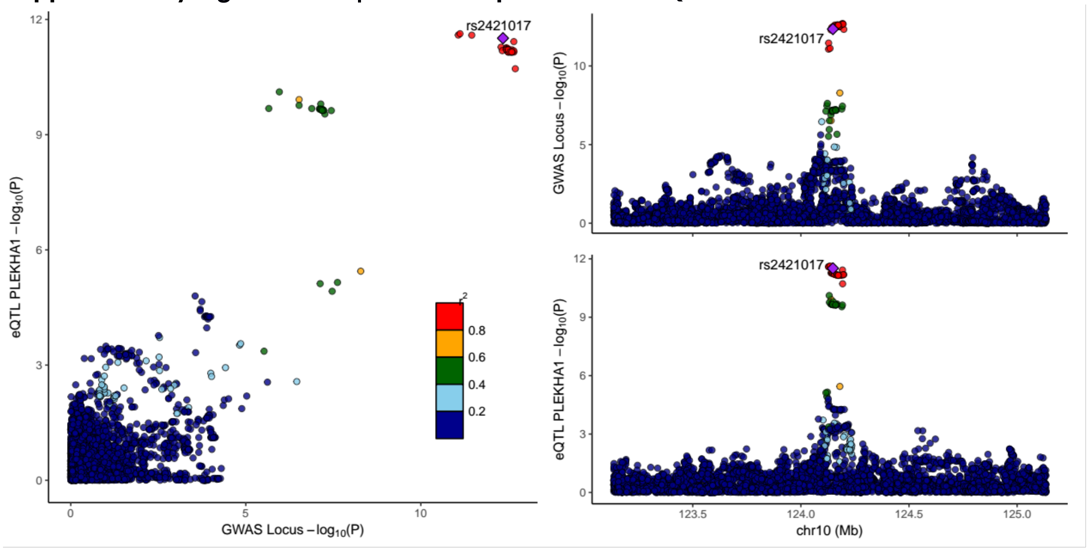

Supplementary Figure 11.23 | LocusCompare for the eQTL in *PLEKHA1* and GWAS loci known as *PLEKHA1*.

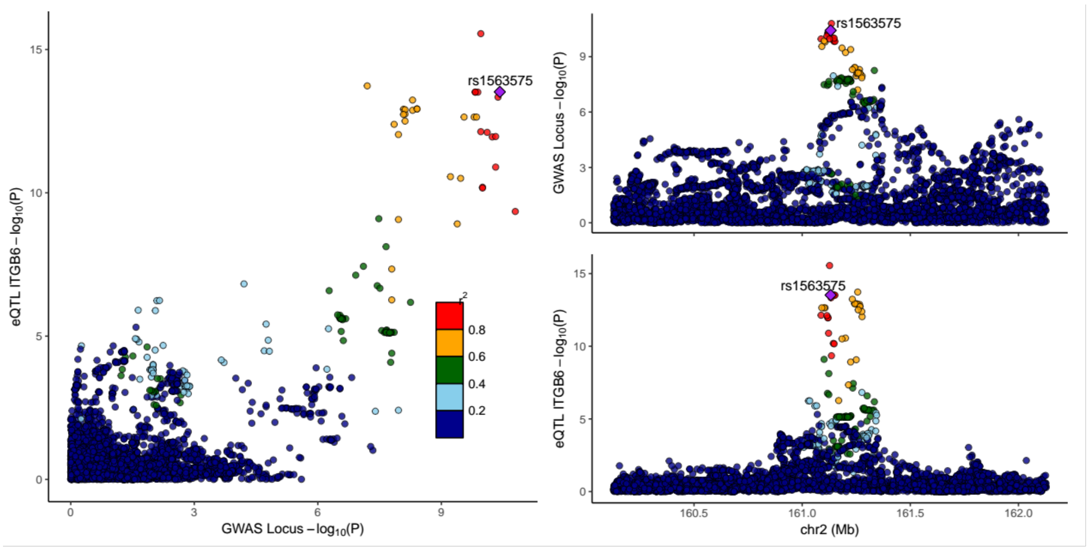

Supplementary Figure 11.24 | LocusCompare for the eQTL in *ITGB6* and GWAS loci known as *RBMS1*.

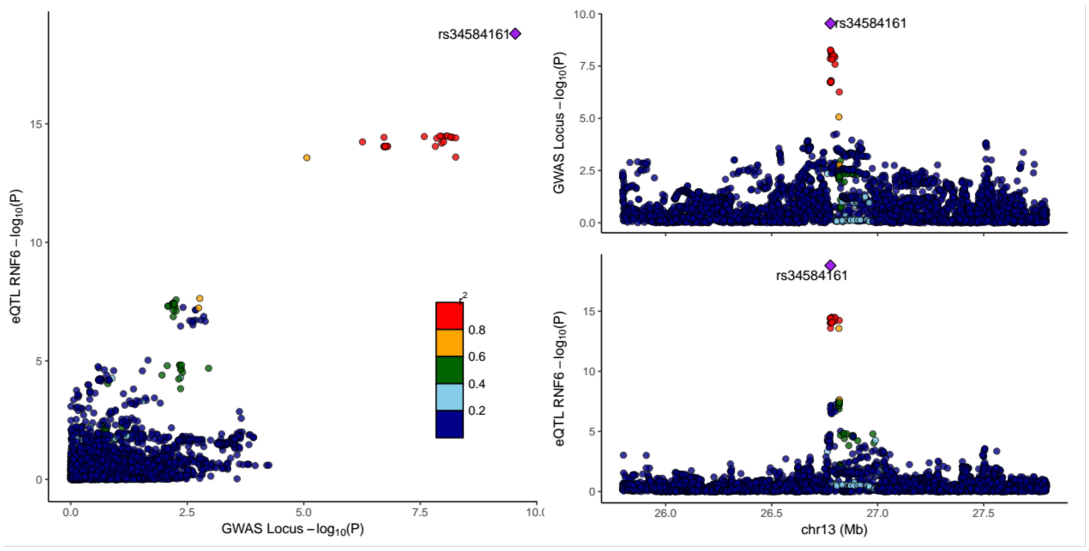

Supplementary Figure 11.25 | LocusCompare for the eQTL in *RNF6* and GWAS loci known as *RNF6*.

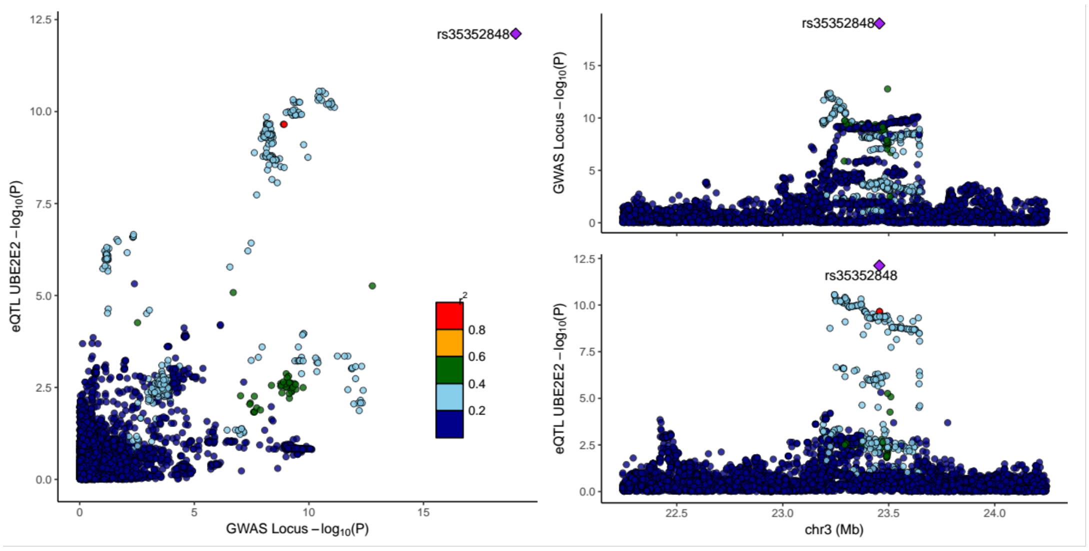

Supplementary Figure 11.26 | LocusCompare for the eQTL in *UBE2E2* and GWAS loci known as *UBE2E2*.

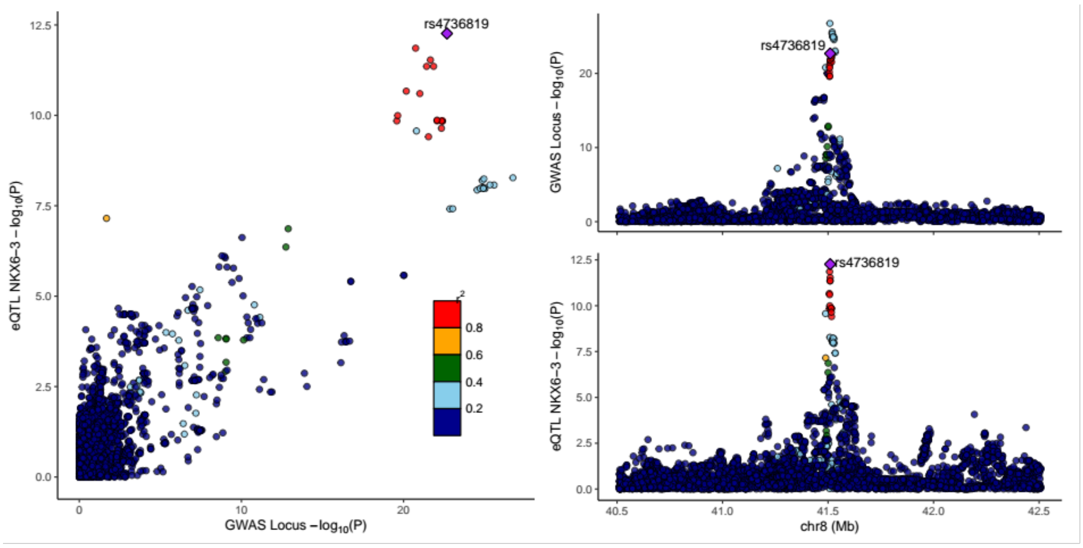

Supplementary Figure 11.27 | LocusCompare for the eQTL in *NKX6-3* and a secondary GWAS loci in *ANK1*.

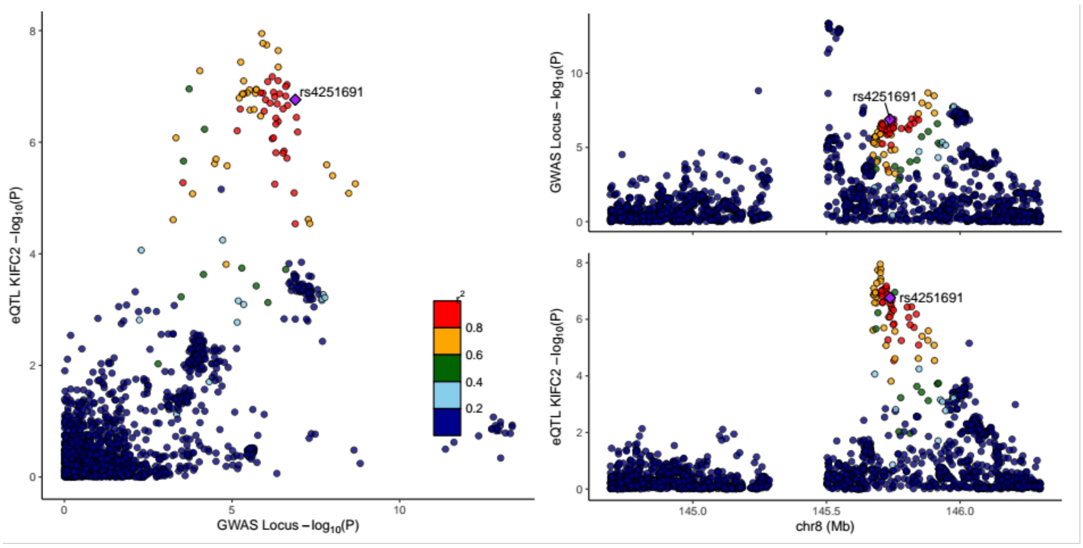

Supplementary Figure 11.28 | LocusCompare for the eQTL in *KIFC2* and GWAS loci known as *BOP1*.

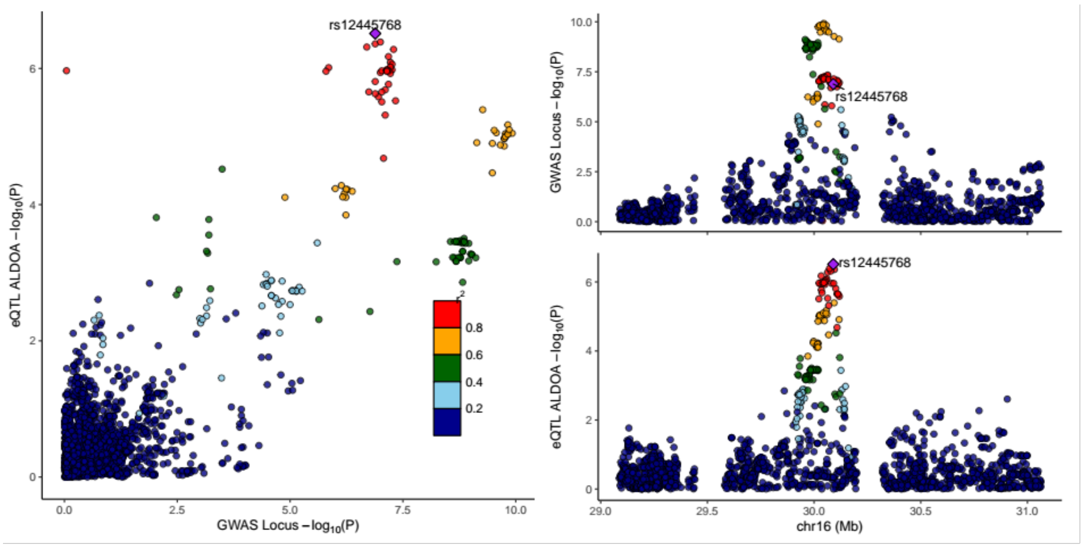

Supplementary Figure 11.29 | LocusCompare for the eQTL in *ALDOA* and GWAS loci known as *FAM57B*.

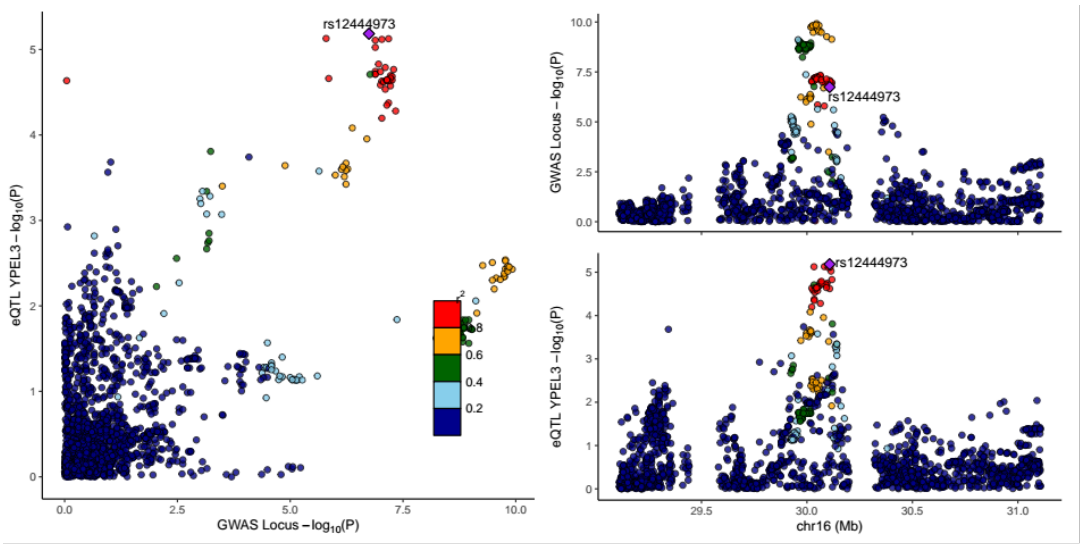

Supplementary Figure 11.30 | LocusCompare for the eQTL in *YPEL3* and GWAS loci known as *FAM57B*.

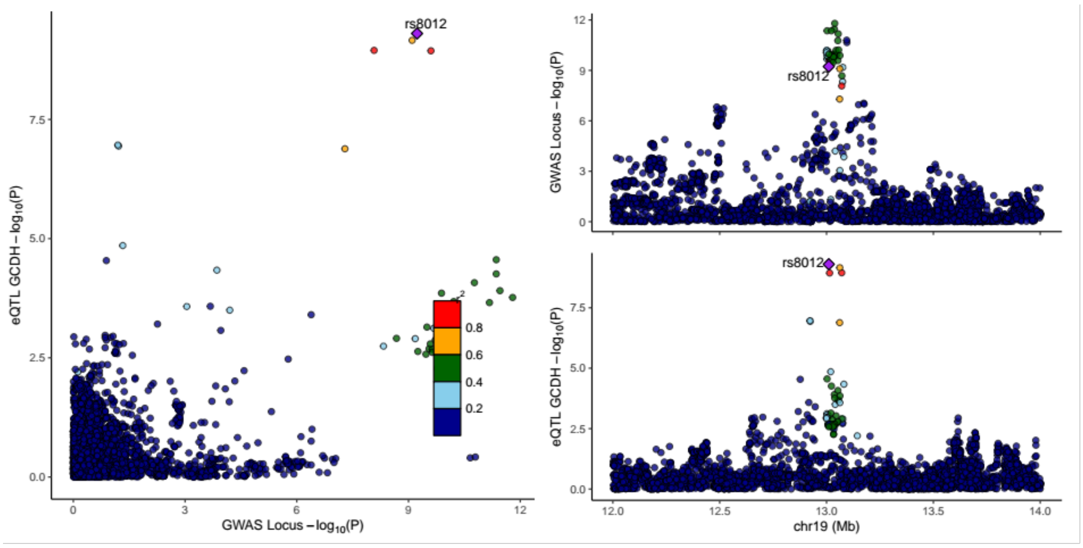

Supplementary Figure 11.31 | LocusCompare for the eQTL in *GCDH* and GWAS loci known as *FARSA*.

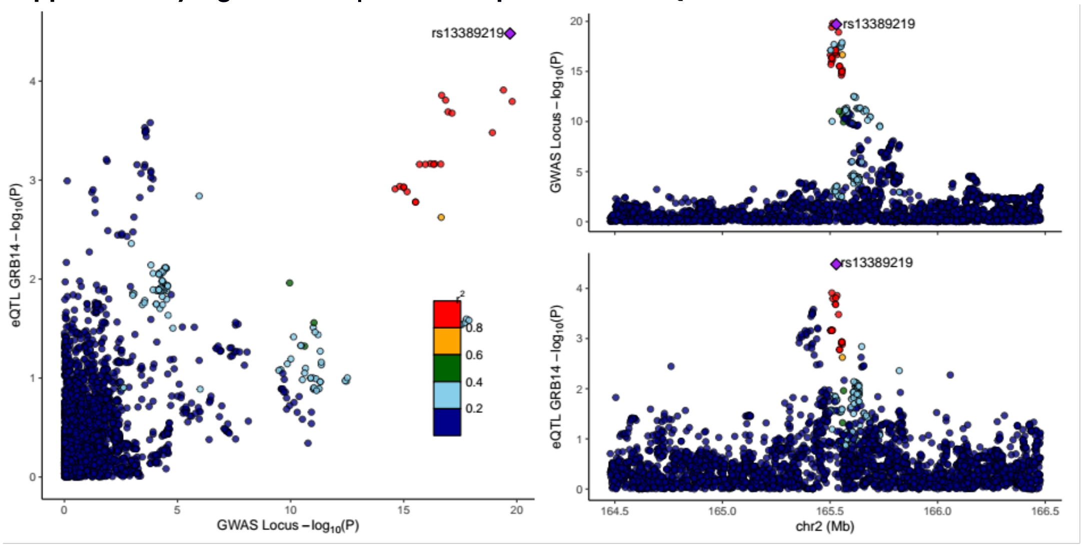

Supplementary Figure 11.32 | LocusCompare for the eQTL in *GRB14* and GWAS loci known as *GRB14/COBLL1*.

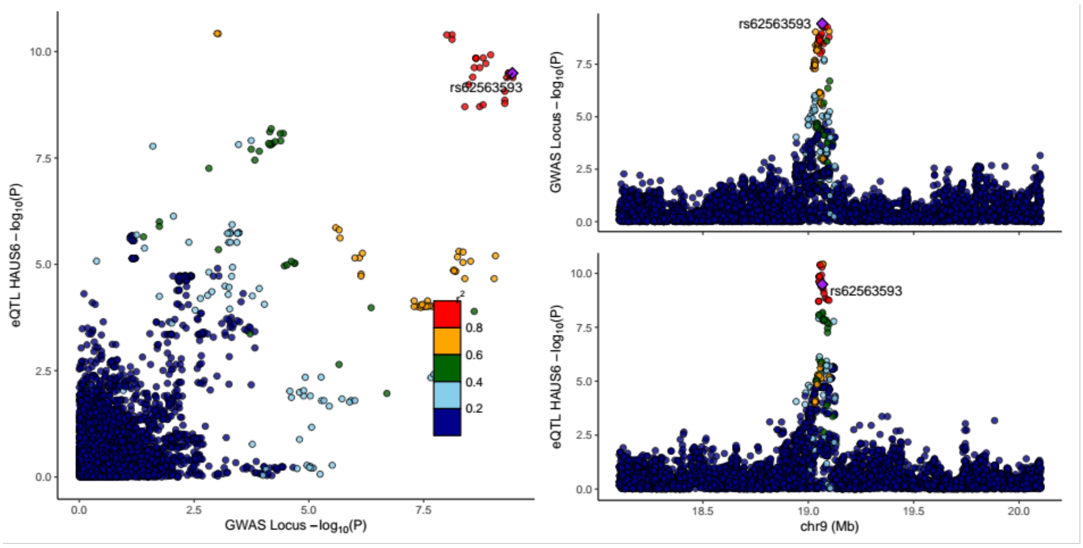

Supplementary Figure 11.33 | LocusCompare for the eQTL in *HAUS6* and GWAS loci known as *HAUS6*.

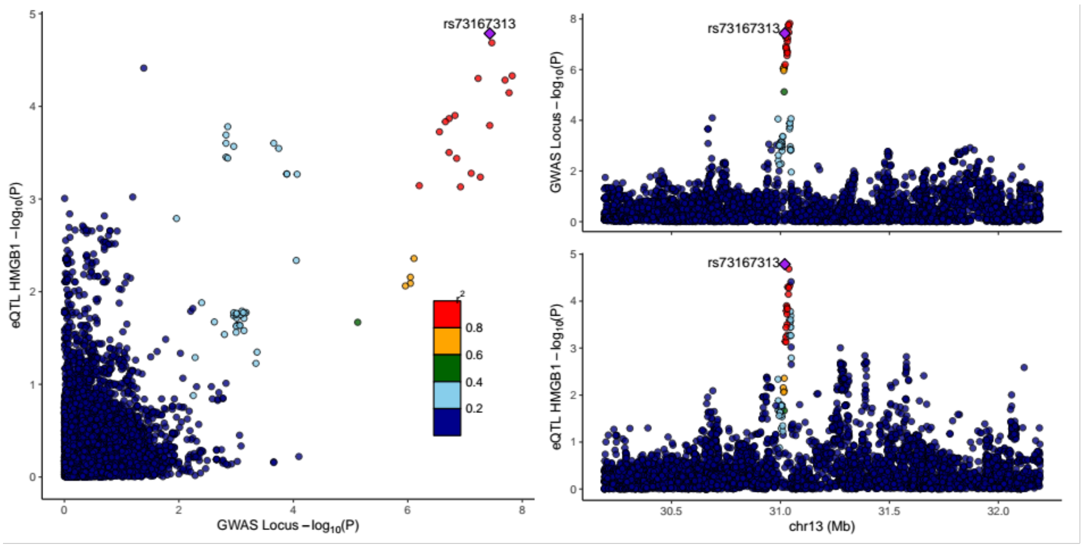

Supplementary Figure 11.34 | LocusCompare for the eQTL in *HMGB1* and GWAS loci known as *HMGB1*.

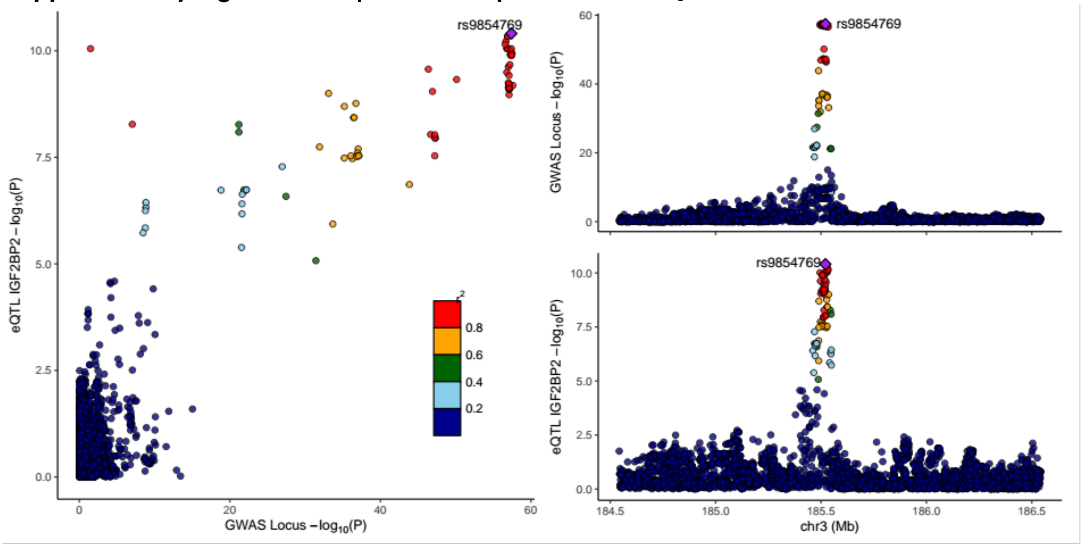

Supplementary Figure 11.35 | LocusCompare for the eQTL in *IGF2BP2* and GWAS loci known as *IGF2BP2*.

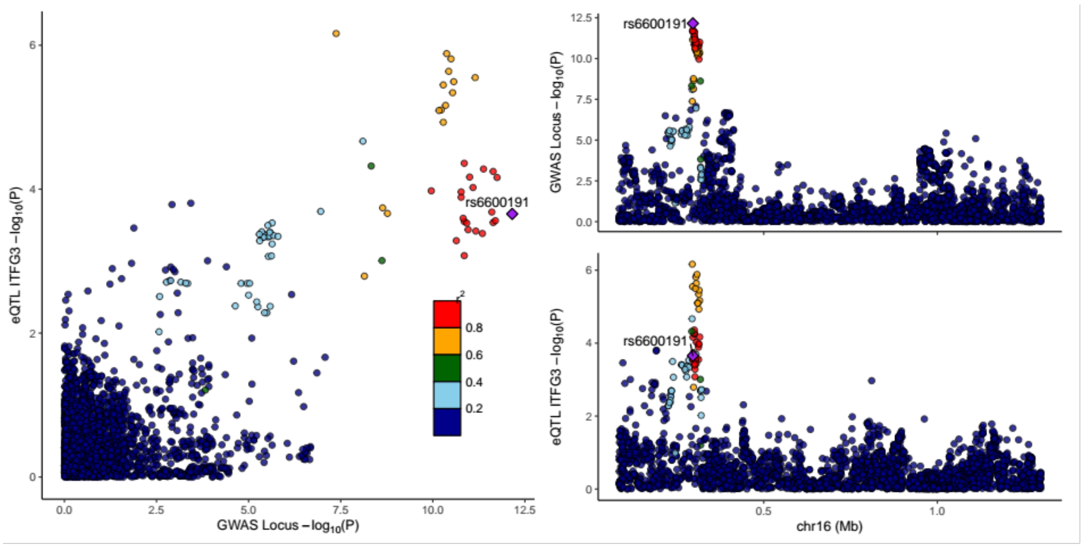

Supplementary Figure 11.36 | LocusCompare for the eQTL in *ITFG3* and GWAS loci known as *ITFG3*.

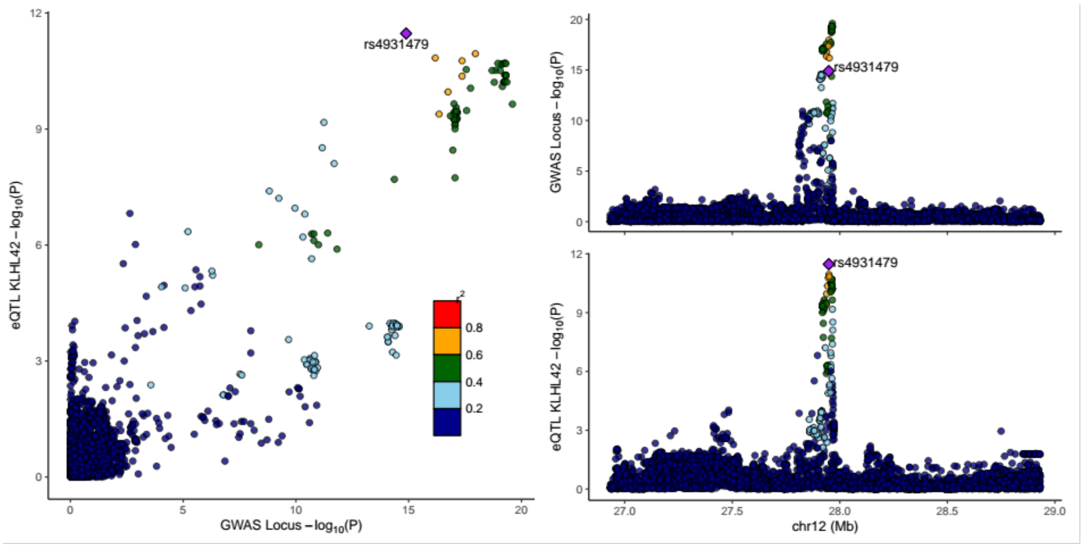

Supplementary Figure 11.37 | LocusCompare for the eQTL in *KLHL42* and GWAS loci known as *KLHDC5*.

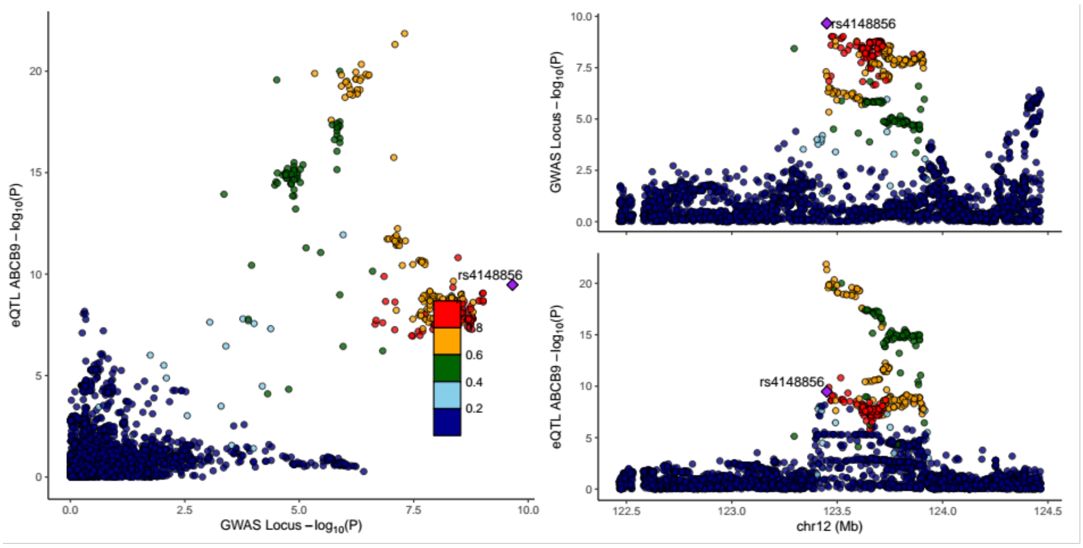

Supplementary Figure 11.38 | LocusCompare for the eQTL in *ABCB9* and GWAS loci known as *MPHOSPH9*.

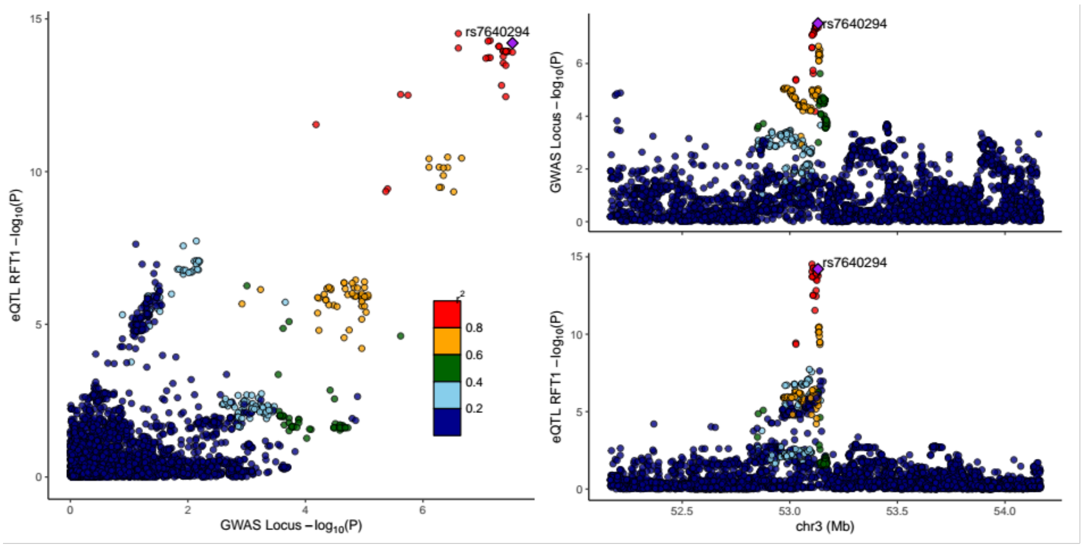

Supplementary Figure 11.39 | LocusCompare for the eQTL in *RFT1* and GWAS loci known as *RFT1*.

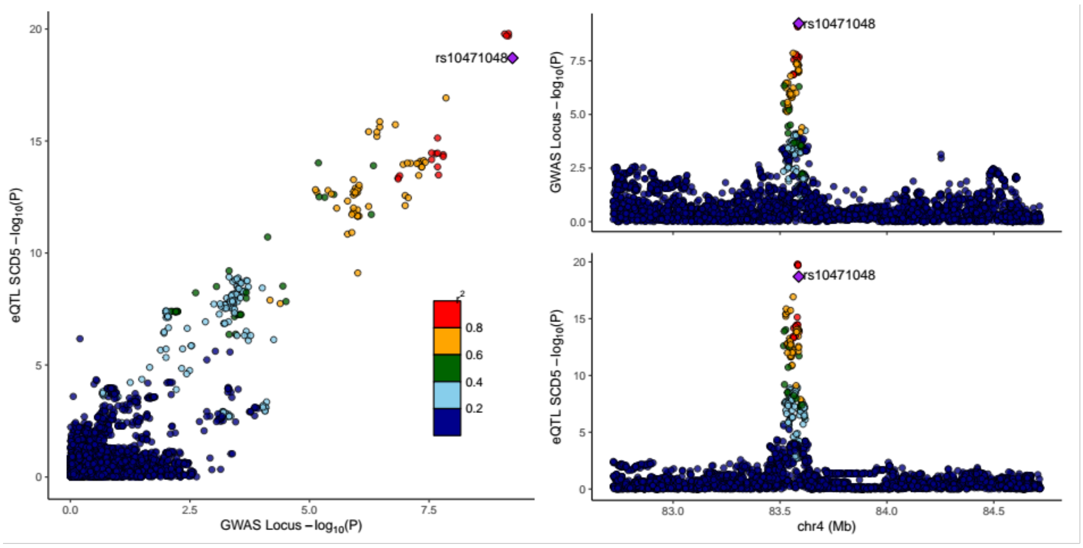

Supplementary Figure 11.40 | LocusCompare for the eQTL in *SCD5* and GWAS loci known as *SCD5*.

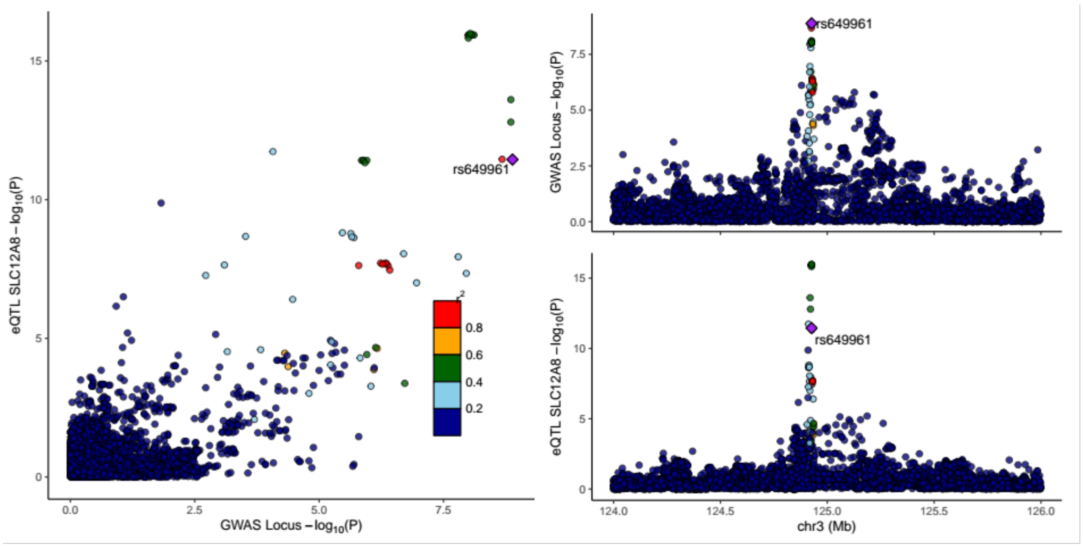

Supplementary Figure 11.41 | LocusCompare for the eQTL in *SLC12A8* and GWAS loci known as *SLC12A8*.

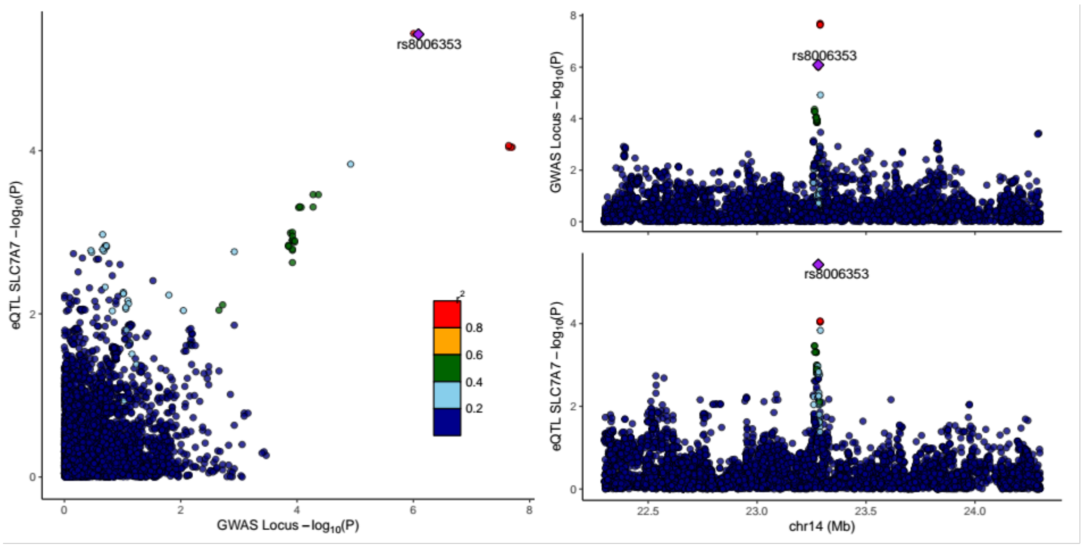

Supplementary Figure 11.42| LocusCompare for the eQTL in *SLC7A7* and GWAS loci known as *SLC7A7*.

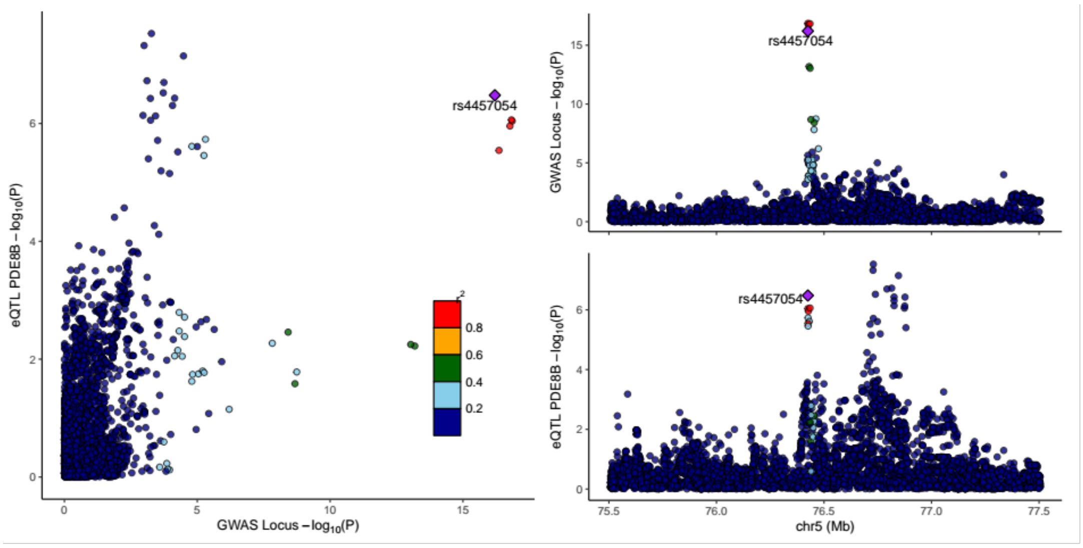

Supplementary Figure 11.43 | LocusCompare for the eQTL in *PDE8B* and GWAS loci known as *ZBED3*.

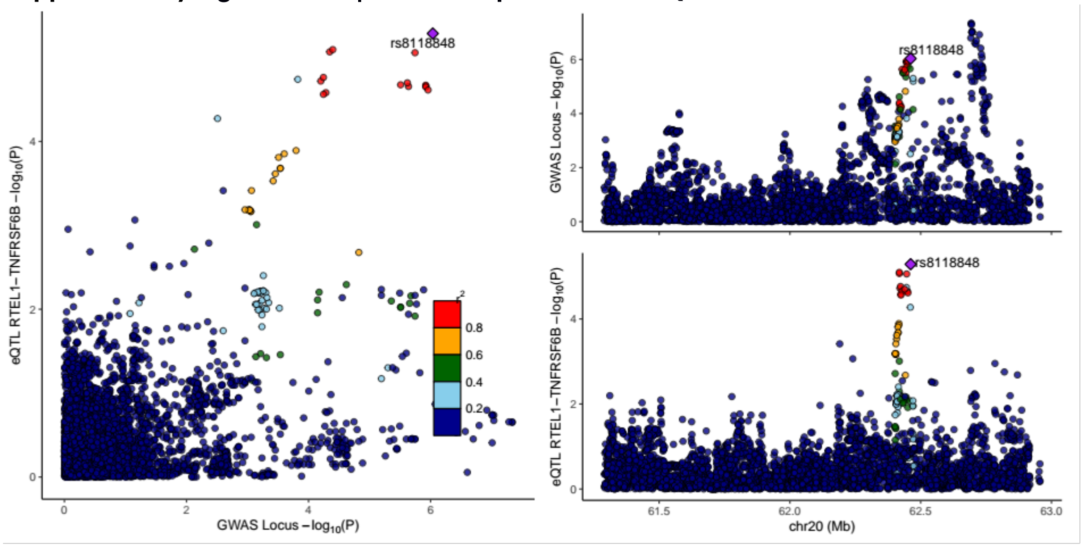

Supplementary Figure 11.44 | LocusCompare for the eQTL in *RTEL1-TNFRSF6B* and GWAS loci known as *ZBTB46*.

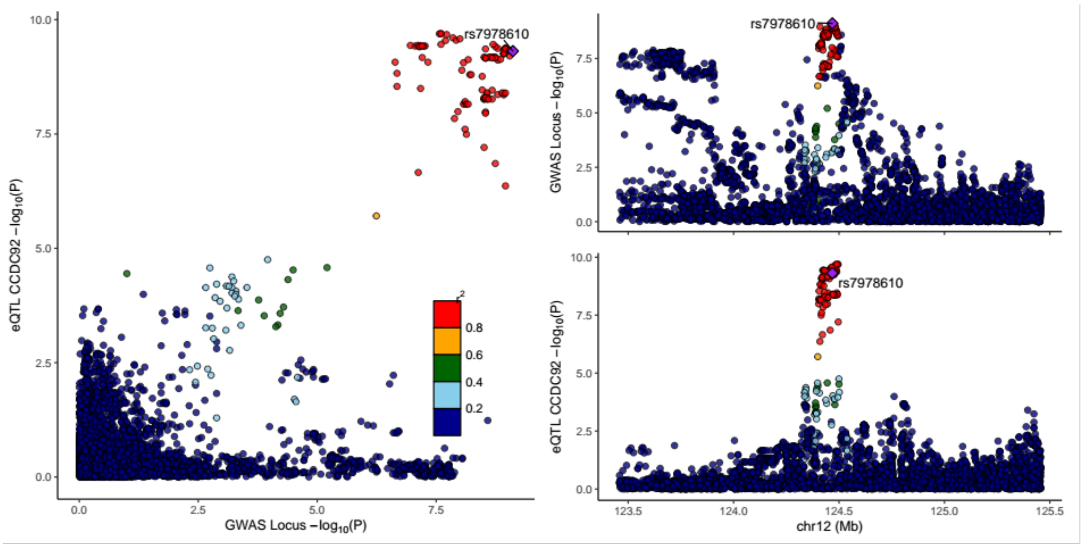

Supplementary Figure 11.45 | LocusCompare for the eQTL in *CCDC92* and GWAS loci known as *ZNF664*.

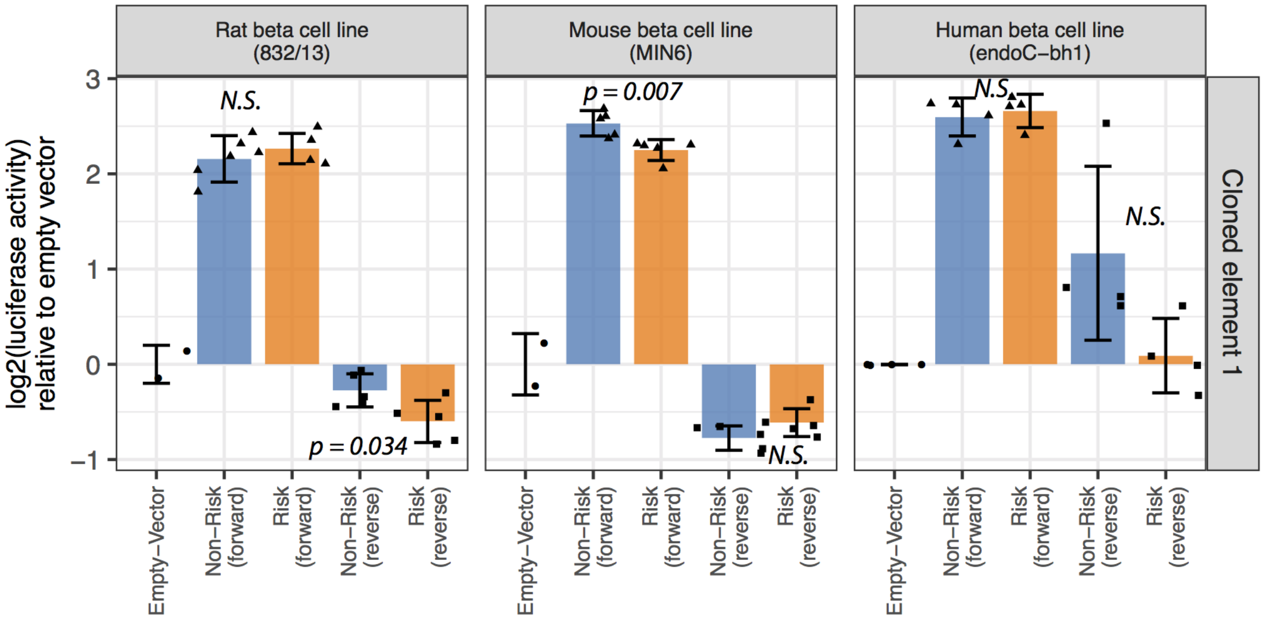

Supplementary Figure 12 | Luciferase assay results for DGKB 3'eQTL element 1: Log 2 Luciferase assay activities (normalized to empty vector) in rat (832/13), mouse (MIN6) and human (endoC) beta cell lines for the element 1 highlighted in green in Figure 3E. The element was cloned in both forward and reverse orientation with respect to the *DGKB* gene. P values were determined using unpaired two-sided t-tests.

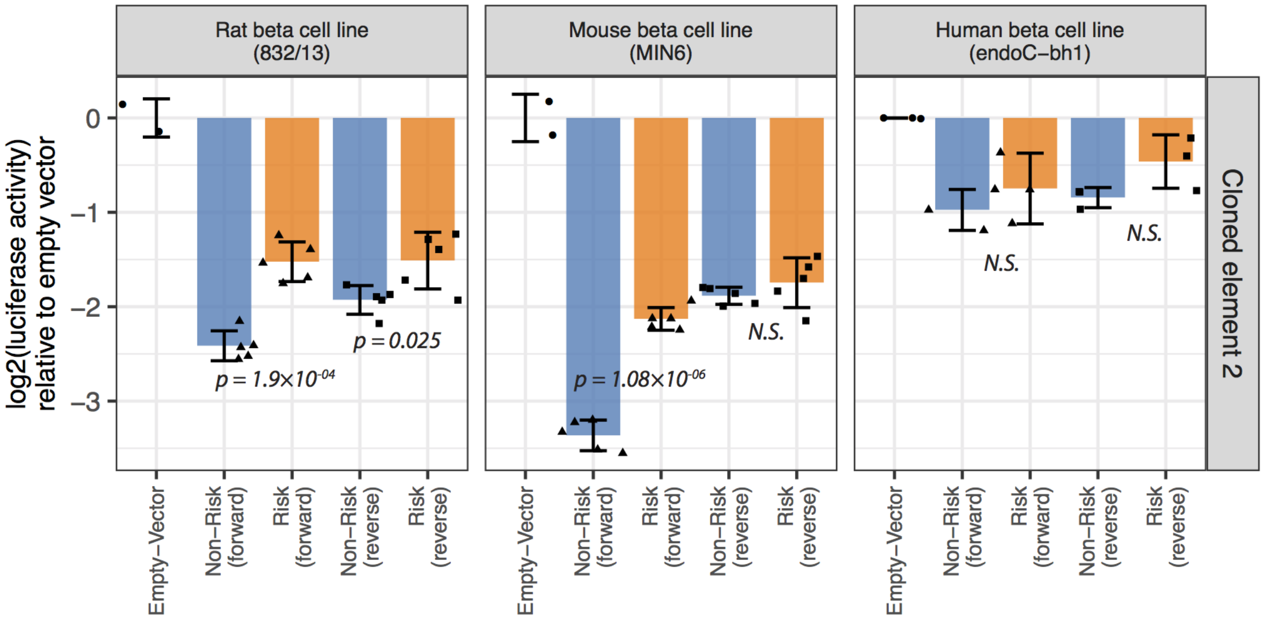

Supplementary Figure 13 | Luciferase assay results for DGKB 3'eQTL element 2: Log 2 Luciferase assay activities (normalized to empty vector) in rat (832/13), mouse (MIN6) and human (endoC) beta cell lines for the element 2 highlighted in blue in Figure 3E, cloned in both forward and reverse orientation with respect to the *DGKB* gene. P values were determined using unpaired two-sided t-tests.

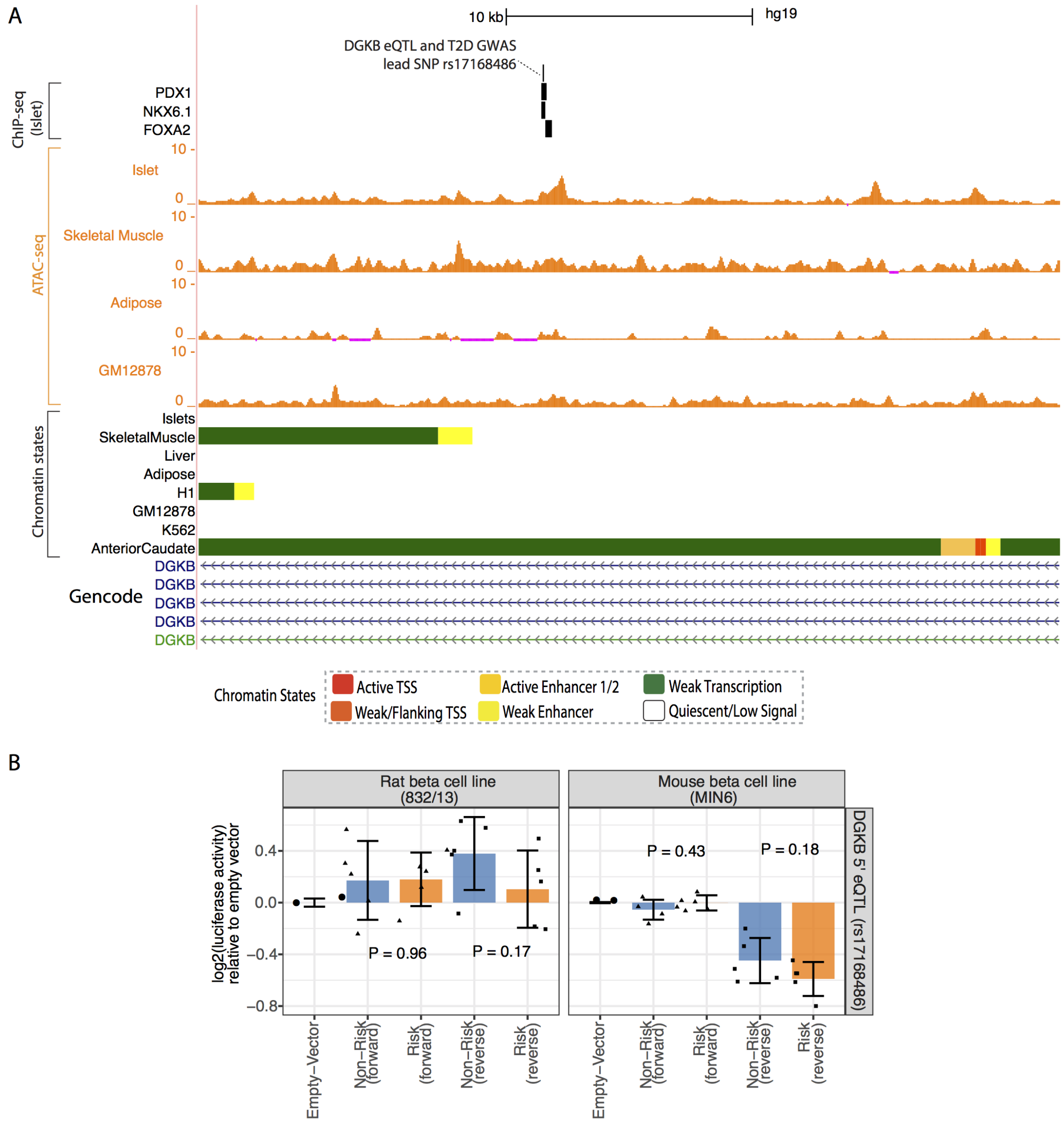

Supplementary Figure 14 | DGKB 5' eQTL locus. 5' eQTL (DGKB eQTL and T2D GWAS lead SNP 17168486). A: Genome browser shot of the 5' DGKB eQTL along with ChIP-seq, ATAC-seq and chromatin state profiles in islets and other tissues. B. Luciferase assay activities (normalized to empty vector) in rat (832/13) and mouse (MIN6) cell lines for the element containing the T2D GWAS and islet eQTL lead SNP (rs17168486), cloned in both forward and reverse orientation with respect to the *DGKB* gene. Differences between activities of the risk and non-risk allele containing elements were non-significant.

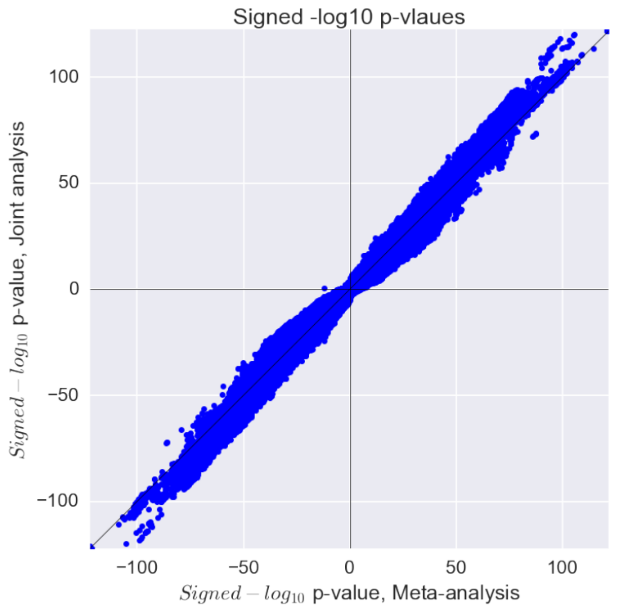

Supplementary Figure 15 | Meta- vs mega-analysis. Comparison or meta-analysis of the four studies versus join re-processing and analysis. A comparison between our joint analyses and a fixed effects meta-analysis of the four studies found highly correlated results indicating appropriate control of the differences across studies.

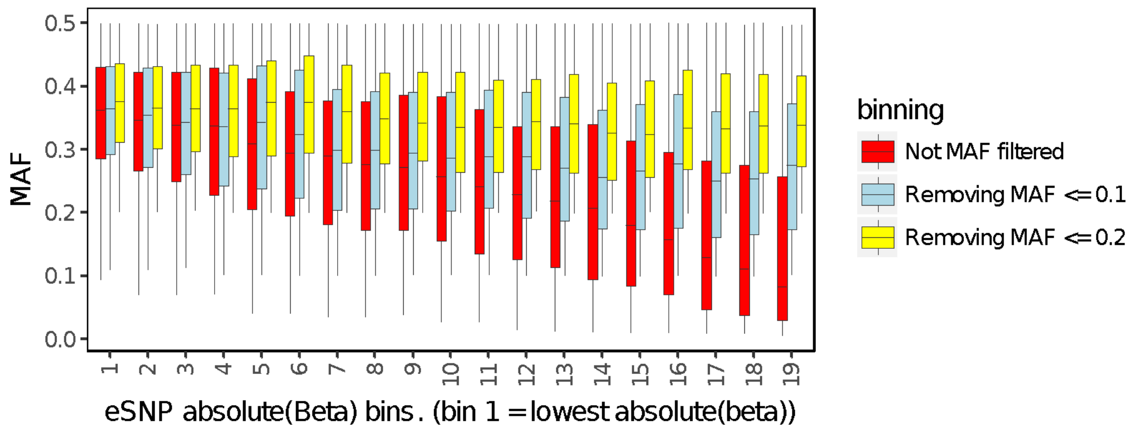

Supplementary Figure 16 | MAF filtering for eSNPs. MAF for islet eQTL eSNPs binned by absolute(beta) into equal sized, 50% overlapping bins. Bin 1 contains eSNPs with lowest absolute(beta), bin19 contains eSNPs with highest absolute(beta).

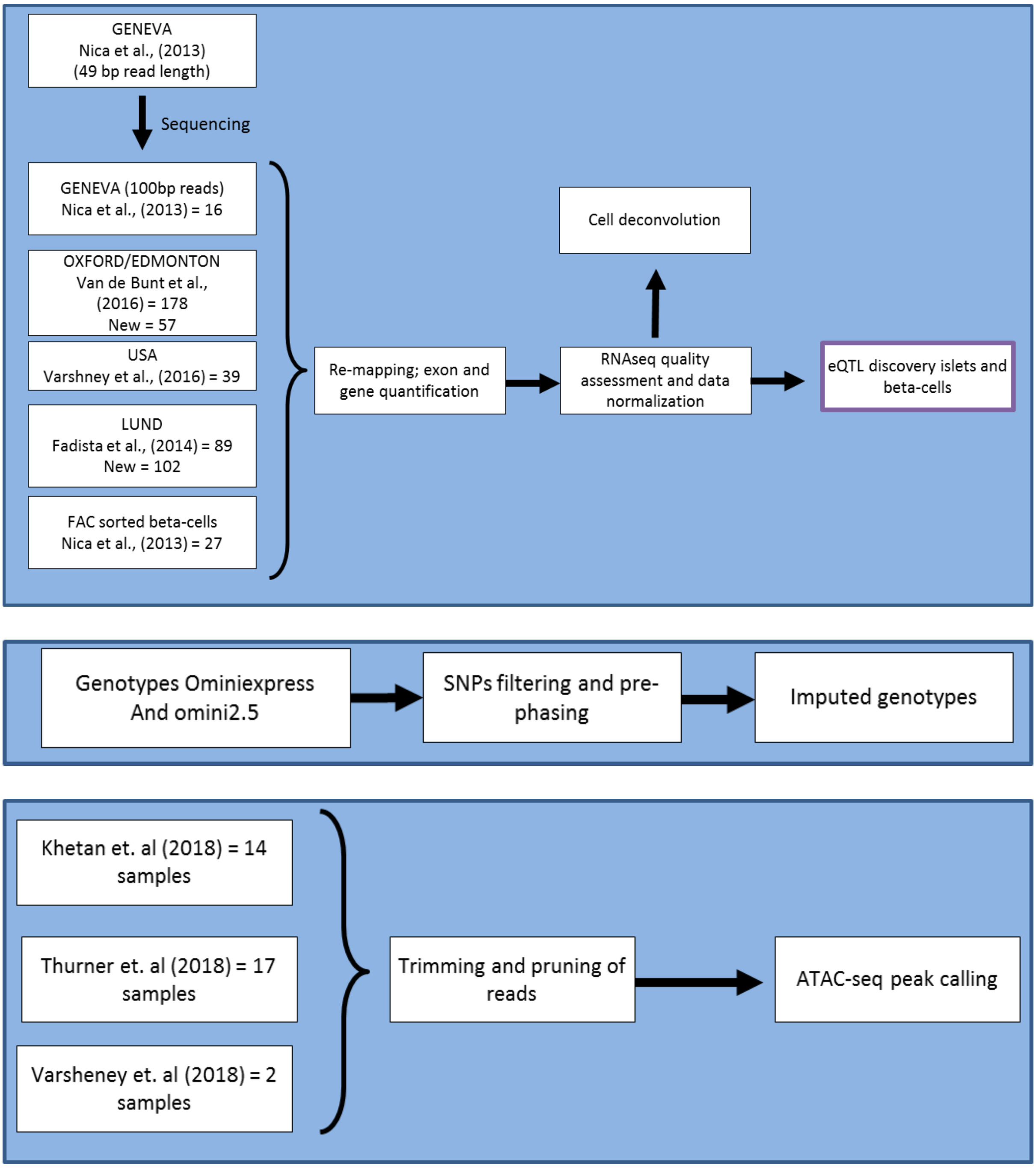

Supplementary Figure 17 | Diagrams showing sample and data processing.

Supplementary References

Aguet, F. *et al.* Genetic effects on gene expression across human tissues. *Nature* **550**, 204 (2017).

Fadista, J. *et al.* Global genomic and transcriptomic analysis of human pancreatic islets reveals novel genes influencing glucose metabolism. *Proc Natl Acad Sci U S A* **111**, 13924-9 (2014).

Rai, V. *et al.* Single-cell ATAC-Seq in human pancreatic islets and deep learning upscaling of rare cells reveals cell-specific type 2 diabetes regulatory signatures. *Molecular Metabolism* **32**, 109-121 (2020).

van de Bunt, M. *et al.* Transcript Expression Data from Human Islets Links Regulatory Signals from Genome-Wide Association Studies for Type 2 Diabetes and Glycemic Traits to Their Downstream Effectors. *PLoS Genet* **11**, e1005694 (2015).

Varshney, A. *et al.* Genetic regulatory signatures underlying islet gene expression and type 2 diabetes. *Proc Natl Acad Sci U S A* **114**, 2301-2306 (2017).
