# Supplementary material for: Genetic variant effects on gene expression in human pancreatic islets and their implications for T2D
Source: Nat Commun. 2020 Sep 30;11:4912. doi: 10.1038/s41467-020-18581-8 (PMC7528108; doi:10.1038/s41467-020-18581-8)
Supplement: Supplementary file 19 — Description of Additional Supplementary Files [file 41467_2020_18581_MOESM19_ESM.docx]

**Description of additional supplementary information**

Supplementary Data 1 = Exon-eQTLs.

Supplementary Data 2 = Genes-eQTLs.

Supplementary Data 3 = Exon-eQTLs not found in gene-eQTLs.

Supplementary Data 4 = Gene-eQTLs not found in exon-eQTLs.

Supplementary Data 5 = eQTLs found only in islets after comparison with GTEx.

Supplementary Data 6 = Beta-cell-eQTLs.

Supplementary Data 7 = Pancreas-eQTLs from GTEx exons.

Supplementary Data 8 = List of 227 beta-cell-eQTLs significant in pancreatic islets.

Supplementary Data 9 = Genotype-by-beta-cells interactions results.

Supplementary Data 10 = Genotype-by-non-beta-lls interactions results.

Supplementary Data 11 = Genotype-by-exocrine-portion interactions results.

Supplementary Data 12 = Binned enrichment of footprints in islets eQTLs.

Supplementary Data 13 = List of GWAS SNPs tested.

Supplementary Data 14 = Results for GTEx tissues enrichment in GWAS variants as eQTLs.

Supplementary Data 15 = GWAS signals colocalizing with islets eQTLs.

Supplementary Data 16 = eQTLs and GWAS overlap with GTI results.
